# miR‐32533 Reduces Cognitive Impairment and Amyloid‐β Overload by Targeting CREB5‐Mediated Signaling Pathways in Alzheimer's Disease

**DOI:** 10.1002/advs.202409986

**Published:** 2025-01-22

**Authors:** Li Zeng, Zhongdi Cai, Jianghong Liu, Kaiyue Zhao, Furu Liang, Ting Sun, Zhuorong Li, Rui Liu

**Affiliations:** ^1^ Institute of Medicinal Biotechnology Peking Union Medical College and Chinese Academy of Medical Sciences Beijing 100050 P. R. China; ^2^ State Key Laboratory of Bioactive Substances and Functions of Natural Medicines Institute of Medicinal Biotechnology Peking Union Medical College and Chinese Academy of Medical Sciences Beijing 100050 P. R. China; ^3^ Department of Neurology Xuan Wu Hospital Capital Medical University Beijing 100053 P.R. China; ^4^ Department of Neurology Baotou Central Hospital Inner Mongolia 014040 P. R. China

**Keywords:** Alzheimer's disease, amyloid‐β peptides, cAMP‐responsive element binding protein 5, microRNA, microRNA‐32533

## Abstract

MicroRNAs (miRNAs) are associated with amyloid‐β (Aβ) dysmetabolism, a pivotal factor in the pathogenesis of Alzheimer's disease (AD). This study unveiled a novel miRNA, microRNA‐32533 (miR‐32533), featuring a distinctive base sequence identified through RNA sequencing of the APPswe/PSEN1dE9 (APP/PS1) mouse brain. Its role and underlying mechanisms were subsequently explored. Bioinformatics and confirmatory experiments revealed that miR‐32533 had a novel 23‐base sequence with minimal coding potential, functioning within the Drosha ribonuclease III (Drosha)/Dicer 1, ribonuclease III (Dicer)‐dependent canonical pathway and identifiable via northern blot. miR‐32533 was abundantly brain‐distributed and downregulated in diverse AD‐related models, including APP/PS1 and five familial AD (5×FAD) mouse brains and AD patient plasma. Overexpression or inhibition of miR‐32533 led to improvements or exacerbations in cognitive dysfunction, respectively, by modulating Aβ production, apoptosis, oxidation, and neuroinflammation through targeting cAMP‐responsive element binding protein 5 (CREB5), which interacted with α disintegrin and metalloproteinase 10 (ADAM10), beta‐site amyloid precursor protein cleaving enzyme 1 (BACE1), and presenilin 1 (PS1) promoters, thereby enhancing Aβ production through BACE1 and PS1 upregulation while suppressing non‐amyloidogenic amyloid precursor protein (APP) processing via ADAM10 downregulation. Furthermore, modulation of the miR‐32533/CREB5 axis ameliorated or worsened cognitive impairment by inhibiting or amplifying Aβ overproduction through the BACE1‐involved amyloidogenic and ADAM10‐involved non‐amyloidogenic pathways. Overall, the findings suggest miR‐32533 as a regulator of Aβ metabolism, oxidative stress, and neuroinflammation, establishing the miR‐32533/CREB5 signaling pathways as potential therapeutic targets for combating Aβ accumulation and cognitive deficits in AD.

## Graphical abstract

microRNA‐32533 (miR‐32533), a novel 23‐base sequence non‐coding RNA, improves Alzheimer's disease (AD)‐like cognitive impairment and inhibits amyloid‐β (Aβ) overload by regulating the beta‐site amyloid precursor protein cleaving enzyme 1 (BACE1)‐involved amyloidogenic and α disintegrin and metalloproteinase 10 (ADAM10)‐involved non‐amyloidogenic pathways through its direct target cAMP‐responsive element binding protein 5 (CREB5) and downstream transcriptional machinery. The novel miR‐32533/CREB5 signaling cascade represents a promising therapeutic avenue to combat Aβ toxicity, oxidative stress, and neuroinflammation in AD.

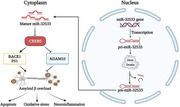

## 1 Introduction

Alzheimer's disease (AD), the predominant neurodegenerative disease affecting the elderly, is characterized by significant dementia alongside cognitive impairment, memory loss, and personality and behavioral alterations. Approximately 50 million individuals worldwide are affected by AD, and projections indicate that this number will increase to 150 million by 2050.^[^ ^1^, ^2^ ^]^ The biological foundation of AD lies in the intracellular accumulation of amyloid‐β (Aβ) and the extracellular deposition of senile plaques, followed by the formation of neurofibrillary tangles derived from hyperphosphorylated tau proteins (p‐tau).^[^ ^3^, ^4^ ^]^ This reciprocal facilitation of Aβ and p‐tau in pathological processes triggers a series of downstream cascades encompassing oxidative stress and neuroinflammation, ultimately leading to neuronal loss, synaptic damage, and comprehensive neurodegeneration with dementia.^[^ ^5^, ^6^ ^]^ Current frontline treatments for AD are acetylcholinesterase inhibitors and N‐methyl‐D‐aspartate receptor antagonists. However, the recent United States (US) Food and Drug Administration (FDA) approval of Aβ‐targeting immunotherapy drugs, Lecanemab (2023) and Donanemab (2024), has sparked interest in amyloid cascade‐based therapeutic strategies.

Dysregulation of Aβ homeostasis is recognized as a driving molecular event in AD. In normal brains, cleavage of amyloid precursor protein (APP) is dominated by the non‐amyloidogenic pathway via α‐secretase (a disintegrin and metalloproteinase 10, *ADAM10*), yielding non‐toxic soluble fragments.^[^ ^7^ ^]^ However, in AD, secretase homeostasis is disrupted, leading to overproduction of Aβ through cumulative cleavage of APP by β‐secretase (beta‐site APP cleaving enzyme 1, *BACE1*) and γ‐secretase (presenilin 1 and 2, *PS1* and *PS2*) in the amyloidogenic pathway.^[^ ^8^ ^]^ As a highly redox‐active peptide, Aβ induces the generation of reactive oxygen species (ROS),^[^ ^9^ ^]^ a critical factor in the propagation of AD pathogenesis. Moreover, Aβ activates brain immune cells, leading to the release of pro‐inflammatory cytokines such as tumor necrosis factor α (TNF‐α) and interleukin 6 (IL‐6), initiating neuroinflammatory responses intricately interwoven with APP processing.^[^ ^10^ ^]^ These oxidative and immunoinflammatory processes serve as crucial links in the promotion of Aβ‐driven vicious cycles and represent promising targets for multi‐pathway therapeutic exploration, which comprises stimulating the α‐secretase‐based non‐amyloidogenic pathway, inhibiting the amyloidogenic pathway of APP processing, and alleviating oxidative stress and inflammation.

microRNAs (miRNAs) are a class of non‐protein transcripts that regulate gene expression at the transcriptional and post‐transcriptional levels based on sequence complementation.^[^ ^11^ ^]^ Their ability to interact with sensitive nodes within deregulated molecular cascades in polygenic disorders renders them attractive candidates for multi‐pathway approaches to AD treatment beyond traditional single‐target therapies. Over 56 miRNAs are implicated in the onset and progression of AD.^[^ ^12^ ^]^ Some miRNAs are directly involved in APP processing and Aβ production, such as miR‐15b, miR‐29c, miR‐124, miR‐135b, miR‐195, and miR‐339‐5p targeting BACE1^[^ ^13^ ^]^ and miR‐30a‐5p, miR‐140‐5p, miR‐144, and miR‐221 targeting ADAM10.^[^ ^14^, ^15^, ^16^ ^]^ Other miRNAs inhibit Aβ‐induced neuronal toxicity, oxidation, or inflammation via regulation of key node proteins of crosstalk cascades, such as miR‐204‐3p mitigating Aβ_1‐42_ toxicity via nicotinamide adenine dinucleotide phosphate oxidase 4^[^ ^17^ ^]^ and an artificial miRNA targeting CD33 molecule (miRCD33) reducing Aβ accumulation and inflammatory response.^[^ ^18^ ^]^ miRNAs are stable in body fluids, and many have been reported to show dysregulation in AD patients.^[^ ^19^ ^]^ However, further investigation is imperative to identify novel disease‐specific miRNAs, comprehend their biological functions, and explore their potential therapeutic application for AD.

In the present study, a previously unknown miRNA with a unique base sequence, termed microRNA‐32533 (miR‐32533), was identified in the brains of APPswe/PSEN1dE9 (APP/PS1) mice using RNA sequencing, quantitative real‐time polymerase chain reaction (qPCR), northern blot, RNA Fluorescence in Situ Hybridization (FISH), and labeling protein encoding analyses. The levels of miR‐32533 were confirmed to be downregulated in various AD models and plasma from AD patients. Functional and mechanistic studies revealed that aberrant miR‐32533 expression influenced cognitive abilities, neuronal apoptosis, oxidative stress, and inflammation by regulating Aβ production via targeting cAMP‐responsive element binding protein 5 (*CREB5*). Further target‐based therapeutic and pathological experiments verified that modulation of the miR‐32533/CREB5 axis ameliorated or aggravated cognitive impairment, along with neurodegeneration, redox imbalance, and neuroinflammation by inhibiting or amplifying Aβ overproduction, respectively, via the BACE1‐involved amyloidogenic and ADAM10‐involved non‐amyloidogenic pathways. Our findings indicate that the novel miR‐32533/CREB5 signaling may serve as a potential therapeutic target for ameliorating Aβ‐elicited cognitive impairment by reducing Aβ production, oxidative stress, and neuroinflammation in AD.

## 2 Results

### 2.1 Identification of miR‐32533 as a Novel miRNA in the Pathological Process of AD

**Figure 1:**
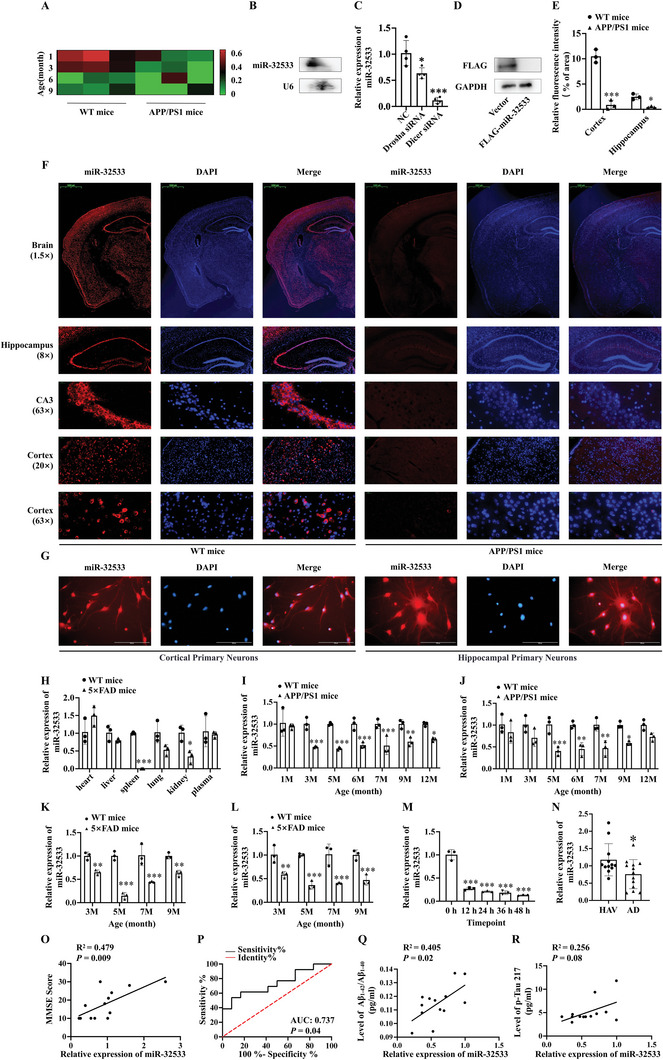
Characterization of the noncoding gene and expression analysis of miR‐32533 in AD pathology. A) RNA‐sequencing analysis of miR‐32533 expression in the cerebral cortex of APP/PS1 mice at 1, 3, 6, and 9 months (*n* = 3), with red indicating a higher relative expression and green indicating a lower relative expression of miR‐32533. B) Northern blot assay detection of miR‐32533 generation in the brains of WT mice. C) Relative expression of miR‐32533 in APPswe cells following Dicer siRNA and Drosha siRNA treatment (*n* = 4). D) Representative Western blot images of FLAG in HEK293 cells transfected with vector p3×FLAG and pre‐miR‐32533‐3×FLAG. E,F) Quantification E) and representative images F) of miR‐32533 levels in WT and APP/PS1 mice using the FISH assay (*n* = 3). Scale bar: 20, 50, 100, or 1000 µm. G) Visualization of miR‐32533 expression in primary mouse hippocampal and cortical neurons by FISH assay. Scale bar: 100 µm. H) Distribution of miR‐32533 across tissues of 5×FAD mice, including heart, liver, spleen, lung, kidney, and plasma (*n* = 3). I,J) qPCR analysis of miR‐32533 expression in the cerebral cortex I) and hippocampus J) of APP/PS1 mice at 1, 3, 5, 6, 7, 9, and 12 months (*n* = 3). K,L) qPCR analysis of miR‐32533 expression in the cerebral cortex K) and hippocampus L) of 5×FAD mice at 3, 5, 7, and 9 months (*n* = 3). M) qPCR analysis of miR‐32533 expression in APPswe cells at 0, 12, 24, 36, and 48 h post‐treatment with 300 µm copper (*n* = 3). N) qPCR analysis of miR‐32533 expression in the plasma of AD patients (*n* = 13) and healthy age‐matched volunteers (HAVs) (*n* = 12). O) Pearson correlation analysis of miR‐32533 plasma expression levels with Mini‐Mental Status Examination (MMSE) scores in AD patients. P) ROC curve analysis for differential miR‐32533 expression in the plasma of AD patients and HAVs. Q,R) Pearson correlation analysis of miR‐32533 expression level with Aβ_1‐42_/Aβ_1‐40_ ratio Q) and p‐Tau217 level R) in the plasma of AD patients. Panels I–L were analyzed using two‐way analysis of variance (ANOVA) followed by Sidak's multiple comparisons test. One‐way ANOVA, followed by Tukey's *post hoc* test, was used for multiple group comparisons to determine differences between groups in panels C, E, H, and M. Two‐tailed Student's *t*‐test was employed for two‐group comparisons in panel N. Results are presented as mean ± standard deviation (SD). ^*^ *p* < 0.05, ^**^ *p* < 0.01, ^***^ *p* < 0.001 versus respective control.

Based on previous RNA‐sequencing data from the brains of APP/PS1 mice (GSE194137) and miRDeep2 software predictions, a novel miRNA with a 23‐base sequence was found to be downregulated in the cerebral cortex (**Figure** **1**A) with a high miRDeep2 score (23.1). This miRNA, designated miR‐32533, was located on mouse chromosome 3 (chr3: 88632253‐88632303: +; Figure S1A, Supporting Information), and its mature sequence was highly conserved across multiple species (Figure S1B,C, Supporting Information), showing a relatively stable precursor stem‐loop structure (Figure S1D, Supporting Information). Northern blot and loss‐of‐function analyses of key enzymes in miRNA biogenesis were performed to understand the role of this novel miRNA. miR‐32533 was detected in the mouse brain (Figure 1B), and silencing of either Drosha ribonuclease III (Drosha) or Dicer 1, ribonuclease III (Dicer) led to significant downregulation of its expression (Figure 1C, *p* < 0.05 vs. negative control (NC)), suggesting miR‐32533 is synthesized in a classical production pathway dependent on Drosha and Dicer. Using bioinformatics analysis, the pre‐miR‐32533 transcript acquired a low coding probability score of 0.004 by the coding potential assessment tool (CPAT) platform, similar to other known miRNAs, such as miR‐23b, miR‐30a, and miR‐148a (Figure S1E, Supporting Information). To further investigate the noncoding potential in vitro, pre‐miR‐32533 was cloned upstream of the p3×FLAG plasmid. Western blotting was performed against the FLAG Tag, indicating no detectable peptides or proteins compared to the p3×FLAG vector (Figure 1D). To examine the spatial expression profiles of miR‐32533, RNA FISH was employed. Our results demonstrated that miR‐32533 exhibited pronounced expression levels in the cerebral cortex and hippocampus of 6‐month‐old wild‐type (WT) mice and primary hippocampal and cortical neuronal cells (Figure 1E–G). However, miR‐32533 expression was significantly decreased in the hippocampus and cortex of APP/PS1 mice (Figure 1E,F, *p* < 0.05 vs. WT mice). In addition, miR‐32533 was expressed in the heart, liver, spleen, lung, kidney, and plasma of five familial AD (5×FAD) mice, as shown by qPCR (Figure 1H). Subsequent quantification of relative copy numbers showed that miR‐32533 had the highest level in the brain of WT mice compared across thirteen distinct tissues examined (Figure S1F, Supporting Information). The potential target genes of miR‐32533 were enriched in multiple biological processes, including metabolic pathways, environmental stress, and neurodegenerative pathways associated with human diseases, including AD, cancers, and infections (Figure S1G, Supporting Information). These results illuminate the properties of the novel miR‐32533 and its underlying role in regulating pathological metabolic processes involved in AD.

Next, qPCR was performed to validate miR‐32533 expression, demonstrating downregulation in the cerebral cortex of APP/PS1 mice between the ages of 3 and 12 months and in the hippocampus from 5 to 9 months, with the lowest expression observed at 5 months (Figure 1I,J, all *p* < 0.05 vs. age‐matched WT mice). Similarly, miR‐32533 expression was significantly downregulated in the cortex and hippocampus of 3‐, 5‐, 7‐, and 9‐month‐old 5×FAD mice, both reaching a minimum at 5 months (Figure 1K,L, all *p* < 0.01 vs. age‐matched WT mice). In addition, miR‐32533 expression was decreased in copper‐triggered APPswe cells from 12 h to 48 h (Figure 1M, all *p* < 0.001 vs. 0 h), as well as in the plasma of AD patients (Figure 1N, *p* < 0.05 vs. healthy age‐matched volunteers (HAVs)). Notably, there was a significant positive correlation between miR‐32533 level and mini‐mental status examination (MMSE) scores in AD patients (Figure 1O, *R^2^* = 0.479, *p* = 0.009). The receiver operating characteristic (ROC) curve analysis indicated a potential diagnostic significance of miR‐32533 in AD patients (Figure 1P and *p* = 0.04). The area under the curve (AUC) for miR‐32533 was 0.737 for AD patients, suggesting a latent diagnostic value with 53.8% sensitivity and 91.7% specificity. Furthermore, a correlation analysis between miR‐32533 and AD diagnostic markers showed that miR‐32533 expression in the plasma of AD patients was correlated with the ratio of Aβ_1‐42_/Aβ_1‐40_ (Figure 1Q and *p* = 0.02), but not significantly correlated with the level of tau phosphorylation at threonine 217 (p‐Tau217) (Figure 1R and *p* = 0.08). Therefore, these results indicate that miR‐32533 is downregulated during AD progression and may be associated with Aβ abnormalities.

### 2.2 miR‐32533 Exerts Neuroprotective Effects and Inhibits Aβ Production In Vitro

**Figure 2:**
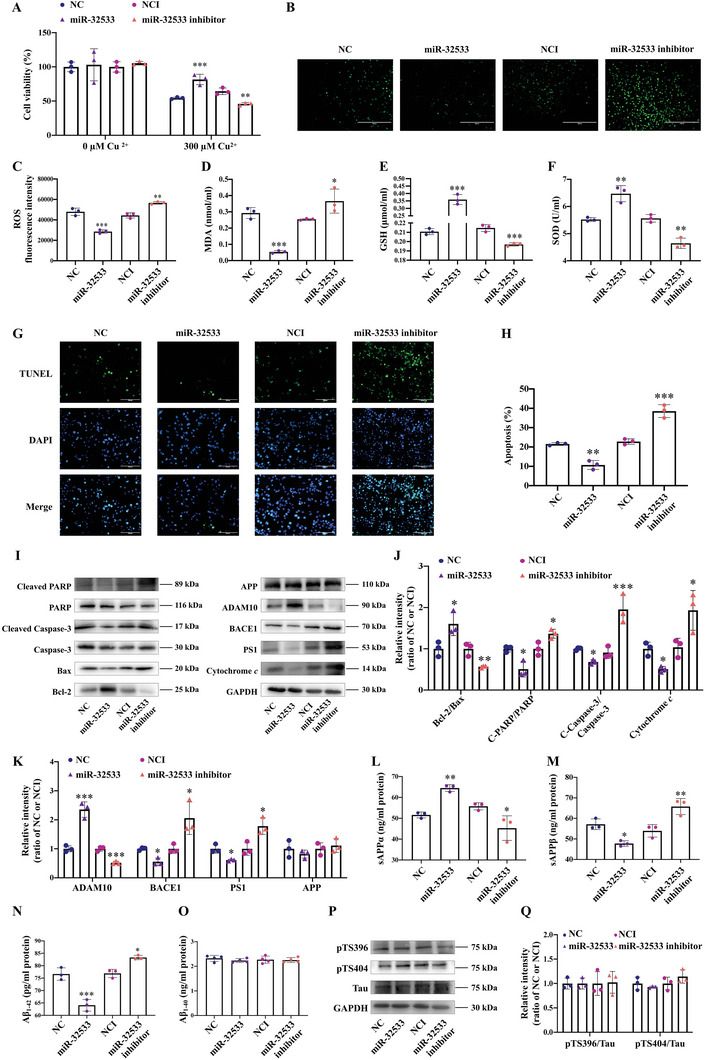
The neuroprotective and Aβ inhibitory effects of miR‐32533 in vitro. A) Cell viability, assessed via MTS assay, in APPswe cells overexpressing or inhibiting miR‐32533, with or without 300 µm copper treatment (*n* = 3). B,C) Representative images B) and mean fluorescence intensity C) of intracellular ROS, stained using 7‐dichlorodihydrofluorescein diacetate (DCFH‐DA), in APPswe cells transfected with miR‐32533 mimics, miR‐32533 inhibitor, and NC/NCI, under 300 µm copper treatment (*n* = 3). Scale bar: 400 µm. D–F) Levels of MDA D), GSH E), and SOD F) in APPswe cells overexpressing or inhibiting miR‐32533 (*n* = 3). G,H) Representative images G) and quantification H) of terminal transferase uridyl nick end labeling (TUNEL)‐positive cells following miR‐32533 overexpression or inhibition in APPswe cells, with 300 µm copper treatment (*n* = 3). Scale bar: 100 µm. I–K) Representative Western blot images I) and quantifications J,K) of PARP, cleaved PARP, caspase‐3, cleaved caspase‐3, cytochrome *c*, Bax, Bcl‐2, ADAM10, BACE1, PS1, APP, and GAPDH in APPswe cells transfected with miR‐32533 mimics, miR‐32533 inhibitor, and NC/NCI (*n* = 3). L–O) Measurements of sAPPα L), sAPPβ M), Aβ_1‐42_ N), and Aβ_1‐40_ O) levels in APPswe cells transfected with miR‐32533 mimics, miR‐32533 inhibitor, and NC/NCI (*n* = 3–4). P,Q) Representative Western blot images P) and quantifications Q) of pTS396, pTS404, Tau, and GAPDH in APPswe cells transfected with miR‐32533 mimics, miR‐32533 inhibitor, and NC/NCI (*n* = 3). Comparisons across multiple groups were analyzed using one‐way ANOVA, followed by Tukey's *post hoc* test to determine intergroup differences. Results are presented as mean ± SD. ^*^ *p* < 0.05, ^**^ *p* < 0.01, ^***^ *p* < 0.001 versus NC/NCI.

To further elucidate the role of miR‐32533 in AD, overexpression and inhibition of miR‐32533 were evaluated by transient transfection of miR‐32533 mimics, miR‐32533 inhibitor, and NC or negative control of inhibitor (NCI) into APPswe cells using copper to trigger Aβ neurotoxicity^[^ ^20^, ^21^, ^22^ ^]^ (Figure S2A,B, *p* < 0.01 vs. NC/NCI). The results showed that miR‐32533 overexpression increased the viability of APPswe cells treated with copper (**Figure** **2**A, *p* < 0.001 vs. NC). Moreover, overexpression of miR‐32533 significantly suppressed ROS production (Figure 2B,C, *p* < 0.001 vs. NC) and malondialdehyde (MDA) level (Figure 2D, *p* < 0.001 vs. NC) and promoted the amount of glutathione (GSH), and superoxide dismutase (SOD) (Figure 2E,F, *p* < 0.01 vs. NC). Oxidative stress has the potential to accelerate the endogenous apoptotic process. As a result, overexpression of miR‐32533 led to significant inhibitory effects on neuronal apoptosis and its associated pathways, as indicated by the reduced apoptotic activity (Figure 2G,H, *p* < 0.01 vs. NC), increased ratio of anti‐apoptotic to apoptotic markers (B cell lymphoma‐2 (Bcl‐2)/Bcl2‐association × protein (Bax)), decreased expression of cytochrome *c*, and lowered ratios of cleaved cysteinyl aspartate‐specific proteinase‐3 (caspase‐3)/caspase‐3 and cleaved poly (ADP‐ribose) polymerase (PARP)/PARP in APPswe cells upon copper stimulation (Figure 2I,J, all *p* < 0.05 vs. NC). However, miR‐32533 inhibition resulted in the opposite effects (Figure 2A–J, all *p* < 0.05 vs. NCI). Overall, these findings suggest that miR‐32533 exerts neuroprotective effects against Aβ‐associated injury.

Furthermore, miR‐32533 overexpression or inhibition significantly increased or decreased ADAM10 expression and suppressed or promoted the expression of BACE1 and PS1 (Figure 2I,K, all *p* < 0.05 vs. NC/NCI) without affecting APP expression. ELISA analysis revealed that miR‐32533 overexpression increased the level of soluble APPα (sAPPα) (Figure 2L, *p* < 0.01 vs. NC), which is involved in the non‐amyloidogenic pathway, and reduced the level of soluble APPβ (sAPPβ) (Figure 2M, *p* < 0.05 vs. NC), which is involved in the amyloidogenic pathway, thereby suppressing Aβ_1‐42_ expression (Figure 2N, *p* < 0.001 vs. NC) without significantly changing Aβ_1‐40_ levels (Figure 2O). However, miR‐32533 inhibition had the opposite effects on the APP processing pathways, resulting in decreased quantities of sAPPα and increased quantities of sAPPβ and Aβ_1‐42_ (Figure 2L–N, all *p* < 0.05 vs. NCI). In terms of tau phosphorylation changes, miR‐32533 overexpression and inhibition showed no significant effect on tau phosphorylation at the Ser404 and Ser396 sites (Figure 2P,Q). Similarly, in SH‐SY5Y cells transiently overexpressing Tau, there were no substantial changes in miR‐32533 expression in response to Tau overexpression (Figure S3A, Supporting Information) and no statistical difference in tau phosphorylation at Ser404 and Ser396 following miR‐32533 overexpression or suppression (Figure S3B,C, Supporting Information). Collectively, these results demonstrate that miR‐32533 plays a neuroprotective role in AD and that its dysregulation may be closely associated with amyloidogenic and non‐amyloidogenic processing for Aβ production.

### 2.3 miR‐32533 Directly Targets CREB5, Which Regulates Key Gene Transcripts in Aβ Production and Oxidative Stress

**Figure 3:**
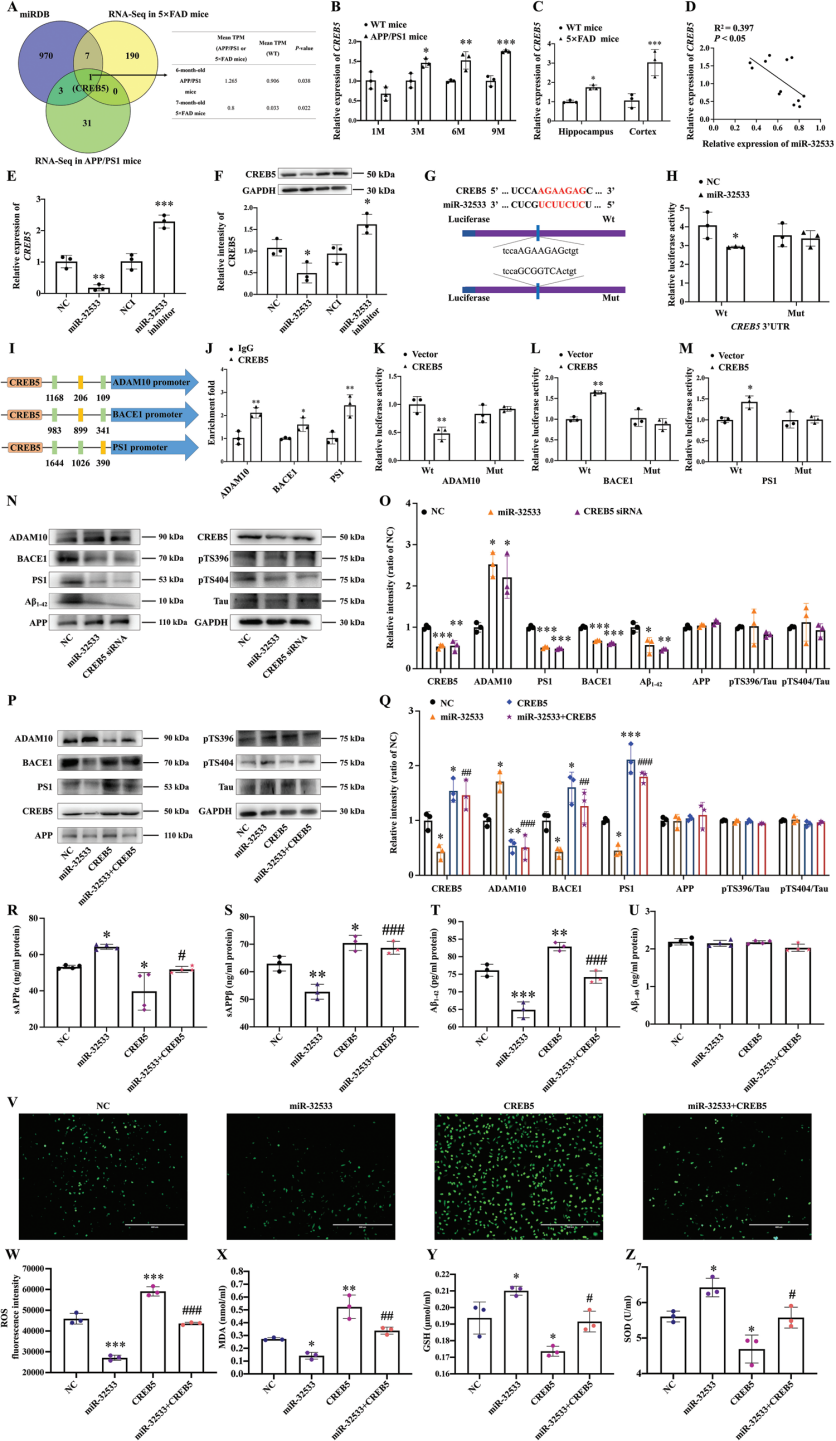
miR‐32533 directly targets CREB5 to regulate Aβ production and oxidative stress. A) Identification of potential target genes of miR‐32533 using miRDB bioinformatics software, coupled with RNA‐sequencing data from the brains of 6‐month‐old APP/PS1 mice and 7‐month‐old 5×FAD mice. B) qPCR analysis of CREB5 levels in the cortex of APP/PS1 mice at 1, 3, 6, and 9 months (*n* = 3). C) qPCR analysis of CREB5 expression in the cortex and hippocampus of 7‐month‐old 5×FAD mice (*n* = 3). D) Pearson correlation analysis between miR‐32533 and CREB5 expression. E) Expression levels of CREB5 in APPswe cells treated with miR‐32533 mimics, miR‐32533 inhibitor, and NC/NCI (*n* = 3). F) Representative Western blot images and quantification of CREB5 protein levels in APPswe cells transfected with miR‐32533 mimics, miR‐32533 inhibitor, and NC/NCI (*n* = 3). G) Bioinformatics analysis of potential binding sites between miR‐32533 and the 3′‐UTR of CREB5, along with a schematic of the construction of dual luciferase reporter gene plasmids, Luc‐CREB5‐Wt and Luc‐CREB5‐Mut. H) Luciferase activity detection in a dual luciferase reporter gene assay, where miR‐32533 was co‐transfected with Luc‐CREB5‐Wt or Luc‐CREB5‐Mut into HEK293 cells (*n* = 3). I) Schematic illustration of potential binding of CREB5 to the promoter regions of ADAM10, BACE1, and PS1. J) ChIP‐qPCR analysis of CREB5‐bound promoter fragments of ADAM10, BACE1, and PS1 extracted from APPswe cells transfected with CREB5. K–M) Relative luciferase reporter activities for ADAM10 K), BACE1 L), and PS1 M) in APPswe cells co‐transfected with CREB5 and corresponding luciferase reporter plasmids. N,O) Representative Western blot images N) and quantifications O) of proteins including ADAM10, BACE1, PS1, Aβ_1‐42_, APP, CREB5, pTS396, pTS404, Tau, and GAPDH in APPswe cells transfected with miR‐32533, CREB5 siRNA, and NC (*n* = 3). P,Q) Representative Western blot images P) and quantifications Q) of proteins including ADAM10, BACE1, PS1, CREB5, APP, pTS396, pTS404, Tau, and GAPDH in APPswe cells transfected with miR‐32533, CREB5, miR‐32533+CREB5, and NC (*n* = 3). R–U) ELISA analysis of sAPPα R), sAPPβ S), Aβ_1‐42_ T), and Aβ_1‐40_ U) levels in APPswe cells transfected with miR‐32533, CREB5, miR‐32533+CREB5, and NC (*n* = 3–4). V,W) Representative images V) and mean fluorescence intensity W) of intracellular ROS in APPswe cells transfected with miR‐32533, CREB5, miR‐32533+CREB5, and NC (*n* = 3). Scale bar: 400 µm. X–Z) Levels of MDA X), GSH Y), and SOD Z) in APPswe cells transfected with miR‐32533 mimics, CREB5, miR‐32533+CREB5, and NC (*n* = 3). Panel B was analyzed using two‐way ANOVA with Sidak's multiple comparisons test. Comparisons among multiple groups were analyzed using one‐way ANOVA, followed by Tukey's post hoc test, employed to analyze differences among multiple groups in panels C, E, F, H, J–M, O, Q–U, and W–Z. Results are presented as mean ± SD. ^*^ *p* < 0.05, ^**^ *p* < 0.01, ^***^ *p* < 0.001 versus NC, ^#^ *p* < 0.05, ^##^ *p* < 0.01, ^###^ *p* < 0.001 versus miR‐32533.

The potential target genes of miR‐32533 in AD were predicted using miRDB bioinformatics software and RNA sequencing datasets. Briefly, 981 target genes of miR‐32533 were obtained using the miRDB online database. In addition, 198 differentially expressed genes were identified in 7‐month‐old 5×FAD mice (GSE206562) and 35 differentially expressed mRNAs were identified in 6‐month‐old APP/PS1 mice (GSE194137). Of these, CREB5, a potential target gene of miR‐32533, was significantly upregulated in both 5×FAD mice (*p* = 0.022) and APP/PS1 mice (*p* = 0.038) (**Figure** **3**A). qPCR further confirmed that CREB5 was significantly upregulated in the cerebral cortex of APP/PS1 and 5×FAD mice (Figure 3B,C, all *p* < 0.05 vs. age‐matched WT mice). miR‐32533 expression was negatively correlated with the CREB5 level (Figure 3D, *p* < 0.05). The miR‐32533 mimics resulted in significantly decreased CREB5 expression at both mRNA and protein levels (Figure 3E,F, *p* < 0.05 vs. NC), while the miR‐32533 inhibitor significantly increased CREB5 mRNA and protein expression levels (Figure 3E,F, *p* < 0.05 vs. NCI). The dual luciferase reporter gene assay showed that miR‐32533 co‐transfected with pGL3‐CREB5‐3′UTR‐Wt significantly reduced luciferase activity in HEK293 cells (Figure 3G,H, *p* < 0.05 vs. NC), while co‐transfected with pGL3‐CREB5‐3′UTR‐Mut had no significant effects on luciferase activity. These findings suggest that miR‐32533 binds directly to CREB5 and negatively regulates CREB5 expression at the transcriptional and translational levels.

Our results showed that miR‐32533 was involved in Aβ metabolic abnormalities by regulating the ADAM10‐mediated non‐amyloidogenic pathway and BACE1/PS1‐mediated amyloidogenic pathway. Given that CREB5 is a transcription factor, we hypothesized its involvement in Aβ metabolism by regulating key gene transcripts, including BACE1, PS1, and ADAM10. The potential binding sites of CREB5 within these genes were predicted using JASPAR software (Table S1, Supporting Information). Subsequent chromatin immunoprecipitation (ChIP)‐qPCR analyses disclosed significant amplification of three promoter segments in APPswe cells (Figure 3I,J): a 206 bp upstream site of ADAM10 promoter (located at chr15: c58751505‐58751488), an 899 bp upstream site of BACE1 promoter (located at chr11: c117317358‐117317347), and a 390 bp upstream site of PS1 promoter (located at chr14: 73134806‐73134818). Other predicted binding sites within these genes failed to show significant enrichment (Figure S4, Supporting Information). To validate the specific interaction of CREB5 with the promoter regions of ADAM10, BACE1, and PS1 and its influence on their transcriptional activities, we constructed their deletion mutation vectors (Mut) and wild‐type vectors (containing binding sites from ChIP‐qPCR analysis). Dual‐luciferase reporter assays showed that in APPswe cells, CREB5 overexpression suppressed ADAM10 transcriptional activity (Figure 3K, *p* < 0.01 vs. Vector), concurrently stimulating the transcriptional activities of BACE1 and PS1 (Figure 3L,M, *p* < 0.05 vs. Vector). Collectively, these results elucidate the role of CREB5 as a novel transcriptional regulator in Aβ metabolism, achieved by directly binding to ADAM10, BACE1, and PS1, thereby suppressing ADAM10 but bolstering BACE1 and PS1 transcription activities.

Subsequently, we conducted further research into the impact of the miR‐32533/CREB5 axis on Aβ metabolism and neuronal oxidative stress. The on‐target and off‐target effect experiments for CREB5 were performed (Figure S5A,B, Supporting Information, both *p* < 0.05 vs. NC/NCI). miR‐32533 overexpression and CREB5 silencing resulted in a decreased Aβ_1‐42_ level by promoting ADAM10 expression and suppressing BACE1 and PS1 expression (Figure 3N,O, all *p* < 0.05 vs. NC). Consistent with the above results (Figure 2P,Q), miR‐32533 overexpression and CREB5 silencing had no significant effects on the phosphorylation of tau protein at the Ser396 and Ser404 sites or APP expression (Figure 3N,O). However, CREB5 overexpression suppressed the levels of ADAM10 (Figure 3P,Q, *p* < 0.01 vs. NC) and sAPPα (Figure 3R, *p* < 0.05 vs. NC), but increased the levels of BACE1, PS1, and sAPPβ (Figure 3P,Q,S, all *p* < 0.05 vs. NC), resulting in an increased Aβ_1‐42_ level (Figure 3T, *p* < 0.01 vs. NC) without a significant change in Aβ_1‐40_ level (Figure 3U). Meanwhile, overexpression of CREB5 antagonized the beneficial effects of miR‐32533 mimics on the upregulation of ADAM10 and sAPPα (Figure 3P–R, both *p* < 0.05 vs. miR‐32533) and the downregulation of BACE1, PS1, and sAPPβ (Figure 3P,Q,S, all *p* < 0.01 vs miR‐32533), resulting in a rebound of the decrease in Aβ_1‐42_ (Figure 3T, *p* < 0.001 vs. miR‐32533). Furthermore, the miR‐32533/CREB5 axis had a role in Aβ‐related oxidative stress, as demonstrated by the reversal effects of CREB5 overexpression on miR‐32533‐mediated reduction of ROS and MDA (Figure 3V–X, *p* < 0.01 vs. miR‐32533) and increase of GSH and SOD (Figure 3Y,Z, *p* < 0.05 vs. miR‐32533). Overall, these results suggest that the miR‐32533/CREB5 axis participates in the pathological processes of Aβ production and oxidative stress.

### 2.4 The miR‐32533/CREB5 Axis is Implicated in AD‐Related Pathologies In Vivo, Including Cognitive Injury, Aβ Production, Oxidative Stress, and Neuroinflammation

**Figure 4:**
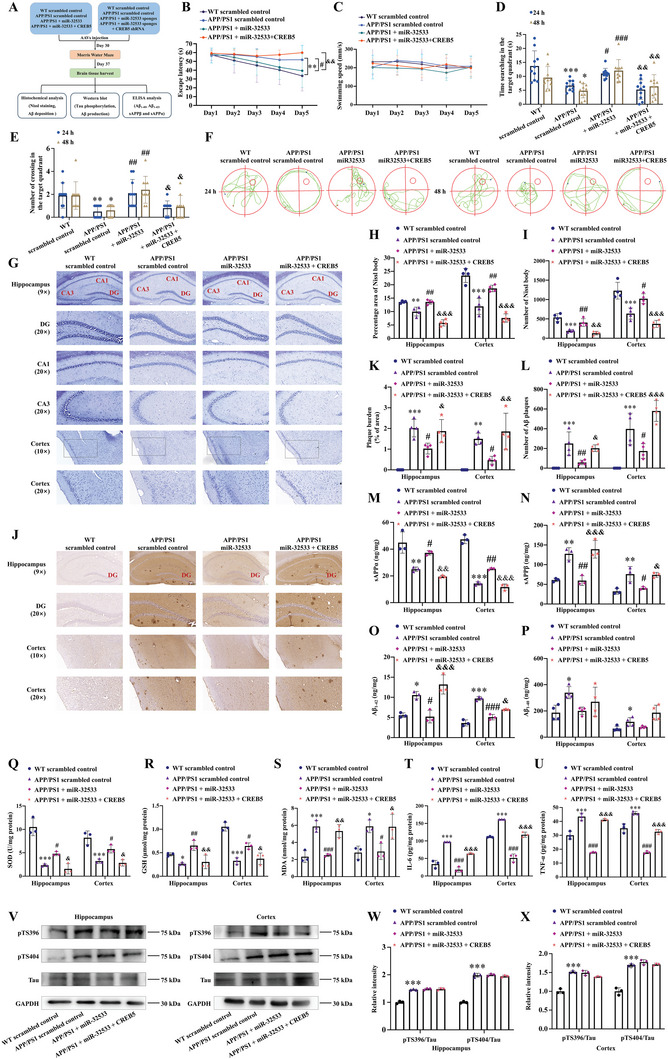
Dependence of miR‐32533 on CREB5 in APP/PS1 mice for improving cognitive ability and altering histopathological features, including neuronal degeneration and Aβ generation. A) Flowchart of mouse enrollment and experimental design. B) Comparative analysis of latency to the platform during five training days in the MWM test for APP/PS1 mice intraventricular infused with miR‐32533 mimics, miR‐32533 mimics plus CREB5, and scrambled sequences (*n* = 10). C) Swimming speed during the navigation test in different treatment groups (*n* = 10). D) Duration spent in the target quadrant by APP/PS1 mice in different treatment groups in the probe test (*n* = 10). E) Number of platform location crossings in the probe test (*n* = 10). F) Representative swimming routes of APP/PS1 mice in different treatment groups on days 6 and 7. G–I) Representative images of Nissl staining G) and quantification H, I) in the hippocampus and cerebral cortex of APP/PS1 mice treated with miR‐32533 mimics, miR‐32533 mimics plus CREB5, and scrambled sequences (*n* = 4). Scale bar: 50 or 100 µm. J–L) Representative images of 6E10 staining J) and quantification K,L) in the hippocampus and cerebral cortex of APP/PS1 mice in different treatment groups (*n* = 4). Scale bar: 50 or 100 µm. M–P) Levels of sAPPα M), sAPPβ N), Aβ_1‐42_ O), and Aβ_1‐40_ P) in the hippocampus and cortex of APP/PS1 mice by ELISA (*n* = 3–4). Q–S) Levels of SOD Q), GSH R), and MDA S) in the hippocampus and cerebral cortex of APP/PS1 mice in different treatment groups (*n* = 3). T and U) Levels of IL‐6 (T) and TNF‐α (U) in the hippocampus and cerebral cortex of APP/PS1 mice in different treatment groups by ELISA (*n* = 3). V–X) Representative Western blot images V) and quantifications W,X) of pTS396, pTS404, Tau, and GAPDH in the hippocampus and cerebral cortex of APP/PS1 mice treated with miR‐32533 mimics, miR‐32533 mimics plus CREB5, and scrambled sequences (*n* = 3). Repeated measures ANOVA was used for panels B and C, while one‐way ANOVA followed by Tukey's *post hoc* test was employed for panels D, E, H, I, K–U, W, and X to analyze group differences. Results are presented as mean ± SD, ^*^ *p* < 0.05, ^**^ *p* < 0.01, ^***^ *p* < 0.001 versus WT scrambled control, ^#^ *p* < 0.05, ^##^ *p* < 0.01, ^###^ *p* < 0.001 versus APP/PS1 scrambled control, ^&^ *p* < 0.05, ^&&^ *p* < 0.01, ^&&&^ *p* < 0.01 versus APP/PS1+miR‐32533.

The therapeutic effect of the miR‐32533/CREB5 axis in vivo was investigated using gain‐ and loss‐of‐function experiments (experimental process shown in **Figure** **4**A). Both miR‐32533 and CREB5 were overexpressed in the brains of APP/PS1 mice after intracerebroventricular injection of adeno‐associated viral (AAV)‐loaded miR‐32533 mimics and CREB5 (Figure S6, Supporting Information, *p* < 0.01 vs. APP/PS1 scrambled control). miR‐32533 mimics improved spatial learning ability of APP/PS1 mice in the Morris water maze (MWM) test, as indicated by reduced platform latencies (Figure 4B, *p* < 0.05 vs. APP/PS1 scrambled control) without swimming speed alterations (Figure 4C). miR‐32533 mimics also ameliorated impairments in hippocampus‐dependent memory, as indicated by a prolonged duration within the target quadrant (Figure 4D, *p* < 0.05 vs. APP/PS1 scrambled control), an increased number of platform crossings (Figure 4E, *p* < 0.01 vs. APP/PS1 scrambled control), and relatively precise and definite travel paths in the probe test (Figure 4F). Notably, overexpression of CREB5 antagonized the protective effects of miR‐32533 mimics on cognitive dysfunction (Figure 4B,D–F, all *p* < 0.05 vs. APP/PS1 + miR‐32533). In addition, there were no significant alterations in escape latency, number of platform crossings, or the duration within the target quadrant in WT mice overexpressing miR‐32533 with or without CREB5, compared to scrambled sequences‐injected WT mice (Figure S7, Supporting Information).

Subsequently, the therapeutic role of the miR‐32533/CREB5 axis in histopathological alterations was assessed by Nissl and 6E10 staining of the hippocampus and cortex of APP/PS1 mice. miR‐32533 mimics mitigated neuronal degeneration, as shown by increased immunoreactive areas and number of Nissl bodies (Figure 4G–I, *p* < 0.05 vs. APP/PS1 scrambled control) and reduced Aβ deposition represented by decreased immunostaining of 6E10 (Figure 4J–L, *p* < 0.05 vs. APP/PS1 scrambled control). However, CREB5 overexpression blocked the protective effects of miR‐32533 mimics on neuronal degeneration and Aβ deposition (Figure 4G–L, all *p* < 0.05 vs. APP/PS1 + miR‐32533).

ELISA analyses further revealed that miR‐32533 mimics promoted the level of sAPPα (Figure 4M, *p* < 0.05 vs. APP/PS1 scrambled control) and inhibited the level of sAPPβ (Figure 4N, *p* < 0.05 vs. APP/PS1 scrambled control), resulting in a reduction of Aβ_1‐42_ (Figure 4O, *p* < 0.05 vs. APP/PS1 scrambled control) with no significant change in Aβ_1‐40_ (Figure 4P). Likewise, CREB5 reversed the regulatory effects of miR‐32533 on sAPPα production (Figure 4M, *p* < 0.01 vs. APP/PS1 + miR‐32533), sAPPβ (Figure 4N, *p* < 0.05 vs. APP/PS1 + miR‐32533), and Aβ_1‐42_ (Figure 4O, *p* < 0.05 vs. APP/PS1 + miR‐32533). CREB5 overexpression also counteracted the promotional effects of miR‐32533 on SOD and GSH contents (Figure 4Q,R, *p* < 0.05 vs. APP/PS1 + miR‐32533), as well as miR‐32533′s inhibitory influence on MDA (Figure 4S, *p* < 0.05 vs. APP/PS1 + miR‐32533), IL‐6, and TNF‐α levels (Figure 4T,U, *p* < 0.001 vs. APP/PS1 + miR‐32533). Overexpression of miR‐32533 and the combination of miR‐32533 and CREB5 did not significantly affect tau protein phosphorylation (Figure 4V–X). These findings indicate that the therapeutic effects of miR‐32533 on AD‐related pathologies, including cognitive impairment, neurodegeneration, Aβ generation, oxidation, and inflammation, may depend on CREB5.

**Figure 5:**
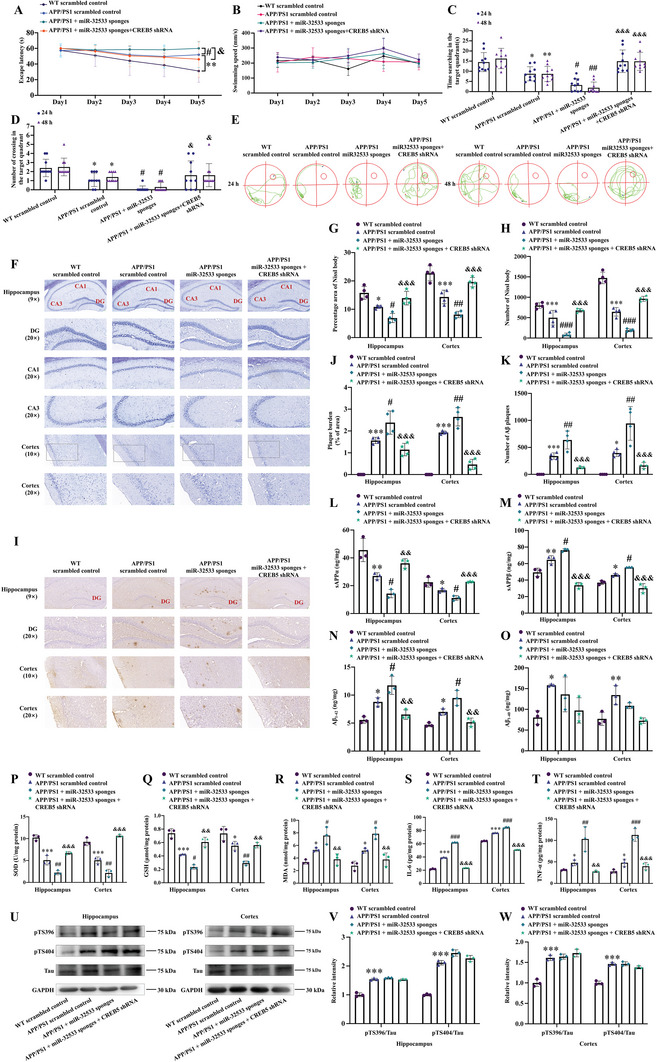
Inhibition of miR‐32533 promotes AD pathologies, including cognitive impairment, neurological injury, and Aβ overproduction, via modulation of CREB5 in APP/PS1 mice. A) Latency in the MWM test during five training days for APP/PS1 mice intraventricular infused with miR‐32533 sponges, miR‐32533 sponges plus CREB5 shRNA, and scrambled sequences (*n* = 10). B) Swimming speed during the navigation test in different treatment groups (*n* = 10). C) Duration within the target quadrant in the probe test (*n* = 10). D) Number of platform location crossings in the probe test (*n* = 10). E) Representative tracing routes of APP/PS1 mice in different treatment groups on days 6 and 7. F–H) Representative images of Nissl staining F) and quantifications G,H) in the hippocampus and cerebral cortex of APP/PS1 mice treated with miR‐32533 sponges, miR‐32533 sponges plus CREB5 shRNA, and scrambled sequences (*n* = 4). Scale bar: 50 or 100 µm. I–K) Representative images of 6E10 staining I) and quantifications J,K) in the hippocampus and cerebral cortex of APP/PS1 mice treated miR‐32533 sponges, miR‐32533 sponges plus CREB5 shRNA, and scrambled sequences (*n* = 4). Scale bar: 50 or 100 µm. L–O) ELISA analysis of sAPPα L), sAPPβ M), Aβ_1‐42_ N), and Aβ_1‐40_ O) levels in the hippocampus and cortex of APP/PS1 mice in different treatment groups (*n* = 3). P–R) Levels of SOD P), GSH Q), and MDA R) in the hippocampus and cerebral cortex of APP/PS1 mice in different treatment groups (*n* = 3). S,T) Levels of IL‐6 S) and TNF‐α T) in the hippocampus and cerebral cortex of APP/PS1 mice by ELISA in different treatment groups (*n* = 3). U–W) Representative Western blot images U) and quantifications V,W) of pTS396, pTS404, and Tau in the hippocampus and cerebral cortex of APP/PS1 mice in different treatment groups (*n* = 3). For panels A and B, repeated measures ANOVA was applied, while one‐way ANOVA was used for panels C, D, G, H, J–T, V, and W, followed by Tukey's *post hoc* test to discern intergroup differences. Results are presented as mean ± SD, ^*^ *p* < 0.05, ^**^ *p* < 0.01, ^***^ *p* < 0.001 versus WT scrambled control; ^#^ *p* < 0.05, ^##^ *p* < 0.01, ^###^ *p* < 0.001 versus APP/PS1 scrambled control; ^&^ *p* < 0.05, ^&&^ *p* < 0.01, ^&&&^ *p* < 0.001 versus APP/PS1+miR‐32533 sponges.

To assess pathogenicity, we investigated the role of the miR‐32533/CREB5 axis in APP/PS1 mice through loss‐of‐function experiments by silencing miR‐32533 and CREB5 in the brain by intracerebroventricular infusion of AAV‐packaged miR‐32533 sponges and CREB5 shRNA (Figure S8, Supporting Information, *p* < 0.001 vs. APP/PS1 scrambled control). As predicted, miR‐32533 sponges exacerbated learning and memory declines in APP/PS1 mice (**Figure** **5**A–E, all *p* < 0.05 vs. APP/PS1 scrambled control). Notably, administration of CREB5 shRNA ameliorated the learning and memory deficits aggravated by miR‐32533 sponges (Figure 5A–E, all *p* < 0.05 vs. APP/PS1 + miR‐32533 sponges). The miR‐32533 sponges also aggravated neuronal degeneration (Figure 5F–H, *p* < 0.05 vs. APP/PS1 scrambled control) and ultimately increased Aβ_1‐42_ production and deposition (Figure 5I–K, all *p* < 0.05 vs. APP/PS1 scrambled control) by further reducing sAPPα (Figure 5L, *p* < 0.05 vs. APP/PS1 scrambled control) and increasing sAPPβ (Figure 5M, *p* < 0.05 vs. APP/PS1 scrambled control) in the hippocampus and cerebral cortex of APP/PS1 mice, leading to exacerbated oxidative stress and inflammatory factor release (Figure 5P–T, all *p* < 0.05 vs. APP/PS1 scrambled control). Similarly, silencing CREB5 ameliorated miR‐32533 inhibition‐induced neuronal degeneration, Aβ_1‐42_ overproduction, redox imbalance, and pro‐inflammatory cytokine generation (Figure 5F–T, all *p* < 0.01 vs. APP/PS1 + miR‐32533 sponges). Consistent with previous observations (Figure 4V–X), no significant changes were observed in tau phosphorylation levels following miR‐32533 and/or CREB5 silencing in APP/PS1 mice (Figure 5U–W). These findings support that CREB5 is involved in miR‐32533 downregulation‐promoted AD pathologies, specifically cognitive injury, abnormal Aβ production, oxidative stress, and neuroinflammation.

### 2.5 The miR‐32533/CREB5 Axis Mediates Aβ Metabolic and Apoptotic Signaling Pathways In Vivo

**Figure 6:**
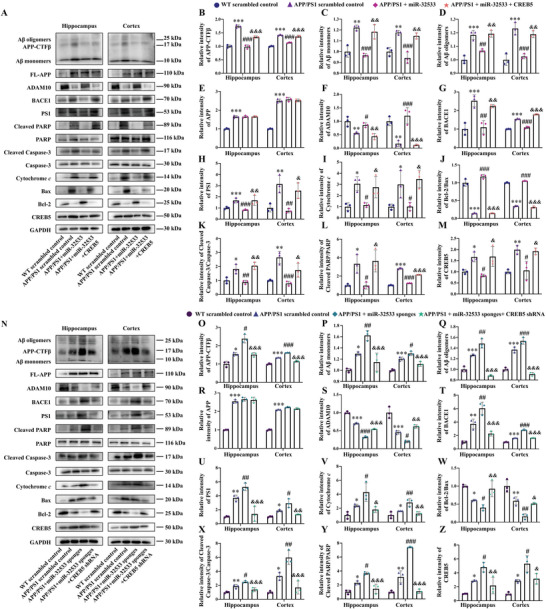
The miR‐32533/CREB5 signaling axis influences APP processing and Aβ production in APP/PS1 mice. A–M) Representative Western blot images A) and quantifications of Aβ (6E10) B–D), APP E), ADAM10 F), BACE1 G), PS1 H), cytochrome *c* I), Bcl‐2/ Bax J), cleaved caspase‐3/ caspase‐3 K), cleaved PARP/ PARP L), and CREB5 M) in the hippocampus and cortex of APP/PS1 mice treated with miR‐32533 mimics, miR‐32533 mimics plus CREB5, and scrambled sequences. N–Z) Representative Western blot images N) and quantifications of Aβ (6E10) O–Q), APP R), ADAM10 S), BACE1 T), PS1 U), cytochrome *c* V), Bcl‐2/Bax W), cleaved caspase‐3/ caspase‐3 X), cleaved PARP/ PARP Y), and CREB5 Z) in the hippocampus and cortex of APP/PS1 mice treated with miR‐32533 sponges, miR‐32533 sponges plus CREB5 shRNA, and scrambled sequences. One‐way ANOVA followed by Tukey's *post hoc* test was used to analyze differences between groups. Results are presented as the mean ± SD. *n* = 3. ^*^ *p* < 0.05, ^**^ *p* < 0.01, ^***^ *p* < 0.001 versus WT scrambled control; ^#^ *p* < 0.05, ^##^ *p* < 0.01, ^###^ *p* < 0.001 versus APP/PS1 scrambled control; ^&^ *p* < 0.05, ^&&^ *p* < 0.01, ^&&&^ *p* < 0.001 versus APP/PS1+miR‐32533 or miR‐32533 sponges.

Informed by the aforementioned in vitro signaling results, we further explored the molecular mechanisms of Aβ metabolism and apoptosis underlying the therapeutic and pathological roles of the miR‐32533/CREB5 axis in vivo. The anti‐Aβ monoclonal antibody 6E10 — which recognizes different forms of Aβ, including full‐length APP, CTFβ, Aβ monomers, Aβ oligomers, and amyloid plaques — was used to explore the influence of the miR‐32533/CREB5 axis on Aβ burden. The miR‐32533 mimics decreased the levels of multiple forms of Aβ, including APP‐CTFβ, Aβ monomers, and oligomers (**Figure** **6**A–D, all *p* < 0.01 vs. APP/PS1 scrambled control), without altering the full‐length APP level (Figure 6A,E) in the hippocampus and cortex of APP/PS1 mice. miR‐32533 mimics also regulated key enzymes in APP processing and apoptotic pathways, as shown by upregulation of ADAM10 expression and downregulation of BACE1 and PS1 levels in these brain regions (Figure 6A,F–H, all *p* < 0.05 vs. APP/PS1 scrambled control), decreased expression of cytochrome *c*, increased Bcl‐2/Bax ratio, and reduced ratios of cleaved caspase‐3/caspase‐3 and cleaved PARP/PARP (Figure 6A,I–L, all *p* < 0.05 vs. APP/PS1 scrambled control). As predicted, miR‐32533 mimics inhibited expression of the target gene CREB5 in the hippocampus and cortex of APP/PS1 mice (Figure 6A,M, both *p* < 0.05 vs. APP/PS1 scrambled control), while CREB5 overexpression reversed miR‐32533‐mediated APP processing and Aβ production, including decreased expression of ADAM10, increased expression of BACE1 and PS1, and elevated levels of APP‐CTFβ, Aβ monomers, and oligomers (Figure 6A–D,F–H, all *p* < 0.05 vs. APP/PS1 + miR‐32533). These results demonstrate that rescue of the miR‐32533/CREB5 axis results in neuroprotection by reducing the Aβ burden via regulation of non‐amyloidogenic and amyloidogenic APP processing pathways in APP/PS1 mice.

Parallel to the findings of miR‐32533 inhibition‐exacerbated cognitive impairment in the APP/PS1 mice, various forms of Aβ, such as monomers, oligomers, and CTFβ, were over‐generated by miR‐32533 sponges (Figure 6N–Q, all *p* < 0.05 vs. APP/PS1 scrambled control), accompanied by a decrease in ADAM10 and an increase in BACE1 and PS1 (Figure 6N,S–U, all *p* < 0.05 vs. APP/PS1 scrambled control). This led to the aggravation of neuronal apoptosis, as indicated by decreased Bcl‐2/Bax ratios and increased cytochrome *c* level, accompanied by elevated cleaved caspase‐3/caspase‐3 and cleaved PARP/PARP ratios (Figure 6N,V–Y, all *p* < 0.05 vs. APP/PS1 scrambled control). As predicted, CREB5 silencing improved the pathological facilitation of Aβ metabolism and burden, as well as APP processing signaling, caused by miR‐32533 inhibition in APP/PS1 mice, ameliorating the apoptotic signaling pathway (Figure 6N–Z, all *p* < 0.05 vs. APP/PS1 + miR‐32533 sponges). Taken together, these results demonstrate the potential role of the miR‐32533/CREB5 axis in APP processing and Aβ production signaling pathways during AD pathological progression.

## 3 Discussion

Extensive research has demonstrated that Aβ burden is a crucial factor in the development of AD, which is interconnected with multifactorial signaling pathways.^[^ ^23^ ^]^ Researchers have been attempting to halt the progression of pathological Aβ biogenesis pathways at the molecular level. However, our research group has taken a unique approach by focusing on specific miRNAs that have the potential to serve as biomarkers and act as molecular nodes in modulating pleiotropic signaling pathways, which may be valuable as therapeutic targets or for gene therapy in the future. In this study, we identified a novel miRNA that targets Aβ metabolism and its pathological damages and subsequently explored its action and molecular mechanisms in AD.

miR‐32533 is a novel sequence that was discovered for the first time in the brains of AD mice using high‐throughput sequencing technology. The miR‐32533 gene shows high conservation across species, featuring a stable precursor stem‐loop structure with low free energy, and it follows the Drosha/Dicer‐mediated classical biogenesis pathway to generate mature miR‐32533. Mature miR‐32533 is 23 nt in length and located at the 5′ end of its precursor stem‐loop structure, indicating the potential for noncoding functionality. Northern blot analysis validated miR‐32533 as a biologically detectable independent transcript in the mouse brain. Notably, miR‐32533 was abundantly expressed in the brain of WT mice, particularly in the areas essential for learning and memory processes, indicating its potential influence on cognitive function. As part of its first pathological description, we consistently observed reduced miR‐32533 expression across various AD models, including APP/PS1 mice, 5×FAD mice, and copper‐triggered APPswe cells, consistent with the RNA‐sequencing outcome. Interestingly, we observed a reduction in miR‐32533 expression to its lowest value in the brains of 5‐month‐old APP/PS1 and 5×FAD mice, whereas its expression slightly increased in 6‐month‐old and older AD mice. During the initial stage of AD, a significant increase in amyloid plaques and inflammatory responses^[^ ^24^, ^25^ ^]^ may lead to the downregulation of miR‐32533. However, as the disease progresses, the rate of increase in amyloid plaques and inflammatory responses levels off.^[^ ^26^ ^]^ Compensatory or protective mechanisms may be activated, leading to a slight recovery in miR‐32533 expression. These observations indicated the potential of miR‐32533 as a biomarker for early diagnosis of AD. Concurrently, we found a decreased level of miR‐32533 in AD patients, which positively correlates with the MMSE score and holds significant clinical diagnostic value. The plasma Aβ_1‐42_/Aβ_1‐40_ ratio and p‐Tau217 level are considered as reference biomarkers for AD, reflecting the extent of senile plaques in the brain^[^ ^27^, ^28^ ^]^ and differentiating AD from other neurodegenerative diseases,^[^ ^29^ ^]^ respectively. Our correlation analysis has revealed that the aberrant expression of miR‐32533 is linked to the changes of Aβ_1‐42_/Aβ_1‐40_ ratio, but not fluctuations in p‐Tau217 level, suggesting a potential role for miR‐32533 in Aβ metabolism during AD progression.

miR‐32533 is closely associated with miR‐6998‐5p, a recently identified miRNA originating from mice in diverse spatial populations through homology analysis. The mmu‐miR‐6998 is situated on mouse chromosome 2 (locus chr2: 31612421… 31612484: +) and can be detected in the brain, bone, heart, and limb muscles (https://www.informatics.jax.org/marker/MGI:5531406). The target genes of miR‐32533 showed significant enrichment in neurodegenerative diseases and metabolism, as shown by the Kyoto Encyclopedia of Genes and Genomes (KEGG) enrichment analysis. Our subsequent experiments demonstrated that miR‐32533 mimics or inhibitor transfected into neuronal cells or administered into mouse brains suppressed or promoted Aβ_1‐42_ production, thereby inhibiting or increasing neuronal apoptosis. Furthermore, miR‐32533 mimics or inhibitor improved or enhanced redox imbalance by modulating antioxidant enzymes (SOD and GSH), peroxides (MDA), and ROS accumulation generated by Aβ‐mediated in vitro neurotoxicity. Given these results, we hypothesized that dysregulation of miR‐32533 in AD might relate to Aβ metabolism and its related pathologies. As predicted, miR‐32533 overexpression in vitro and in vivo increased ADAM10 and sAPPα levels in the non‐amyloidogenic pathway, while reducing BACE1, PS1, and sAPPβ levels in the amyloidogenic pathway, decreasing Aβ_1‐42_ production in gain‐of‐function experiments. In contrast, loss‐of‐function experiments revealed that miR‐32533 inhibition had opposite effects on the expression of the above‐mentioned molecules involved in Aβ metabolic pathways. The use of AAV vectors to alter target genes for gene therapy has been approved by the US FDA. In our functional experiments, AAV‐loaded miR‐32533 mimics were constructed and used to rescue learning and memory deficits shown by AD mice in the MWM test and reduced Aβ deposition in brains from the BACE1/PS1‐involved amyloidogenic pathways. In contrast, the use of AAV‐constructed miR‐32533 sponges worsened cognitive impairment and cerebral Aβ loading. Additional functional evidence suggests that miR‐32533 is capable of reducing the excessive generation of Aβ, mitigating oxidative stress and neuroinflammation, and ultimately improving cognitive deficits.

Another key finding of this study is the potential mechanism through which miR‐32533 influences Aβ production via its gene target, *CREB5*. *CREB5*, which is located on chromosome 7 (7p15.1) and belongs to the activating transcription factor/CREB family, encodes a transcriptional activator in eukaryotic cells.^[^ ^30^ ^]^ At present, the majority of studies on the role of CREB5 are centered on tumors,^[^ ^31^, ^32^ ^]^ infections,^[^ ^33^, ^34^ ^]^ and certain degenerative diseases such as idiopathic achalasia^[^ ^35^ ^]^ and Parkinson's disease.^[^ ^36^ ^]^ A study showed that CREB5 was significantly upregulated in the frontal cortex of patients with human immunodeficiency virus encephalitis,^[^ ^37^ ^]^ suggesting that CREB5 may play a vital role in neuroinflammation. Previous studies have reported that CREB5 contributes to the regulation of redox balance and inflammatory mediator production by acting as a pivotal component in the TNF signaling pathway.^[^ ^38^, ^39^ ^]^ To date, several miRNAs have been reported to modulate tumor sensitivity, malignancy, and metastasis by targeting CREB5, including miR‐142‐3p, miR‐150‐5p, miR‐342‐3p, miR‐132‐3p, miR‐211‐5p, miR‐204, and miR‐206.^[^ ^40^, ^41^, ^42^, ^43^, ^44^ ^]^ Recent reports have indicated that CREB5 expression is upregulated in late‐onset AD, and this upregulation intensifies the interrelation of CREB5 with cerebral atrophy and Aβ deposition in the entorhinal cortex in a genome‐wide transcriptome analysis.^[^ ^45^ ^]^ Our study offers experimental evidence to support the role of CREB5 in AD, as well as its genetic regulation and gene transcription mechanisms.

Our results suggest CREB5 working as a pathogenetic factor in AD, which showed a preferable impact on Aβ metabolism, rather than tau hyperphosphorylation, in vitro. Subsequent findings indicate that acquisition or depletion of CREB5 increased or decreased the Aβ_1‐42_ level via regulation of ADAM10‐implicated sAPPα and BACE1/PS1‐implicated sAPPβ productive pathways. Through specific binding to the corresponding promoter regions, CREB5 effectively inhibited the transcriptional activity of ADAM10, a key protease involved in the non‐amyloidogenic process, and promoted the transcriptional activities of BACE1 and PS1, two core enzymes in the amyloidogenic pathway. As a result, the activation of CREB5 exacerbates the oxidative stress and neuroinflammation induced by excessive Aβ production, promotes neuronal apoptosis, and ultimately accelerates AD progression. Thus, the CREB5 function was elucidated in orchestrating the transcriptional landscape of key players in Aβ metabolic dysregulation, making it a key factor for the ameliorative role of miR‐32533 in AD.

**Figure 7:**
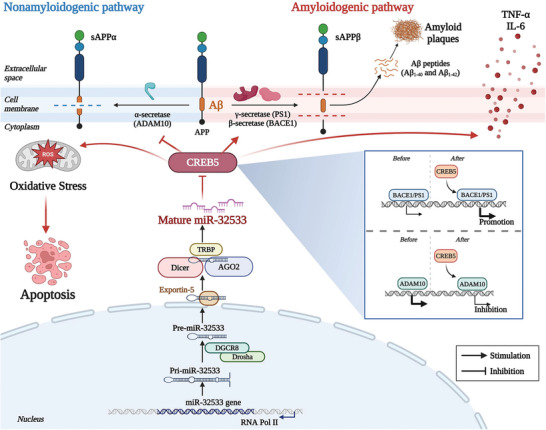
The proposed mechanism underlying the miR‐32533/CREB5/Aβ signaling pathways, based on the findings of the present study. Aβ: amyloid‐β; ADAM10: a disintegrin and metalloproteinase 10; AGO2: Argonaute 2; APP: amyloid precursor protein; BACE1: beta‐site APP cleaving enzyme 1; CREB5: cAMP‐responsive element binding protein 5; DGCR8: DGCR8 microprocessor complex subunit; Dicer: Dicer 1, ribonuclease III; Drosha: Drosha ribonuclease III; IL‐6: interleukin 6; PS1: presenilin‐1; RNA pol II: RNA polymerase II; sAPPα: soluble APPα; sAPPβ: soluble APPβ; TNF‐α: tumor necrosis factor α; TRBP: TARBP2 subunit of RISC loading complex. (Created using BioRender.com).

Overexpression or inhibition of miR‐32533 resulted in down‐ or up‐regulation of CREB5 in the hippocampus and cerebral cortex of APP/PS1 mice. In addition to the repressive effects of miR‐32533 on CREB5 expression at the transcriptional and translational levels, the pathological changes and associated signaling transduction induced by CREB5 were also modulated by miR‐32533 mimics or inhibitors, including excessive Aβ production, oxidative imbalance, neuroinflammation, and neuronal apoptosis. This suggests a potential connection between miR‐32533 and CREB5 signaling in AD. Furthermore, the pathological phenotypes of AD caused by miR‐32533 overexpression or silencing in APP/PS1 mice, such as spatial cognition, neurodegeneration, oxidative stress, and inflammation, were altered by co‐transfection with CREB5 or CREB5 shRNA. Parallel to these findings, the beneficial effects of miR‐32533 on the molecular cascades of APP processing by promoting the non‐amyloidogenic pathway and inhibiting the amyloidogenic pathway occurred in a CREB5‐dependent manner. Therefore, these findings establish a potential link for miR‐32533/CREB5/Aβ signaling in experimental models (**Figure** **7**).

While this study has laid a foundation for understanding the function of this novel miRNA, there are still some interesting questions that warrant further exploration. First, large samples of clinical patients need to be recruited to clarify the clinical significance of miR‐32533. Second, considering the wide distribution in various tissues of miR‐32533 in APP/PS1 mice, it is necessary to investigate its time‐, space‐, and tissue‐specific characteristics in different diseases. In addition, miR‐32533 may have many different targets in the brain; other biological functions of miR‐32533, including Aβ‐induced neuroinflammation, and in vivo tau pathology, are yet to be determined.

## 4 Conclusion

In conclusion, our study presents the initial evidence regarding the role and potential mechanisms of miR‐32533 in AD pathogenesis. miR‐32533 ameliorates AD‐like cognitive impairment and suppresses the accumulation of Aβ from monomers and oligomers to extracellular plaques by modulating the ADAM10/sAPPα processing in the non‐amyloidogenic pathway and BACE1/PS1/sAPPβ signaling in the amyloidogenic pathway, which is attributed to the targeting of CREB5 via its transcription mechanisms. These tentative findings suggest the potential of the miR‐32533/CREB5/Aβ signaling axis as a novel therapeutic target for mitigating Aβ toxicity, oxidative stress, and neuroinflammation in AD, highlighting the significance of miRNA‐based therapeutic strategies.

## 5 Experimental Section

### Animals and Treatments

APP/PS1 transgenic mice with a C57/BL6 background and their littermate WT mice were purchased from Zhishan (Beijing) Institute of Health Medicine Co., Ltd. (Beijing, China). 5×FAD mice were obtained from Changzhou Cavens Model Animal Co., Ltd. (Jiangsu, China). All in vivo animal procedures were authorized by the Animal Ethics Committee of the Institute of Medicinal Biotechnology, Chinese Academy of Medical Sciences (IMB‐20230221D7).

### Cell Culture

APPswe cells at passage numbers fewer than 15 were used to imitate the pathologic specificity of Aβ hyper‐deposition by overexpressing the Swedish‐type APP gene in human neuroblastoma SH‐SY5Y cells (American Type Culture Collection, CRL‐2266). APPswe cells were treated with 300 µm copper sulfate pentahydrate (abbreviated as copper, sourced from BioRuler, Danbury, USA), which forms chelates with Aβ and induces excessive Aβ deposition. These cells were routinely cultured in Dulbecco's Modified Eagle's Medium (DMEM) supplemented with 10% fetal bovine serum (FBS) and 1 µg mL^−1^ puromycin (Beyotime Biotechnology, Shanghai, China), imitating the pathological features of Aβ accumulation in AD.

Primary mouse cortical and hippocampal neurons were isolated from newborn C57/BL6 mice at postnatal day 1.^[^ ^46^ ^]^ After meticulous removal of the meninges and superficial blood vessels, the cerebral cortex and hippocampus were dissected. These tissues were gently clamped with fine forceps and subjected to a 25‐min digestion process using 0.25% trypsin at 37 °C. Digestion was terminated with DMEM complete medium containing 10% FBS, and the tissue suspension was blown and mixed, sieved, and centrifuged at 1000 rpm for 5 min. The cell suspensions were resuspended in Neurobasal‐A complete medium, enriched with 2% B27, 1% glutamine, and 1% penicillin‐streptomycin. Subsequently, the cells were seeded into culture flasks pre‐coated with Poly‐L‐lysine. The following experiments commenced 10 days after plating.

### Cell Transfection

The miR‐32533 mimics, antisense miR‐32533 oligonucleotides (acting as the miR‐32533 inhibitor), NC, and NCI were synthesized by GenePharma (Shanghai, China). The pcDNA3.1‐Tau, pcDNA3.1‐CREB5, and CREB5 siRNA were obtained from Sangon Biotech Co., Ltd. (Shanghai, China). The plasmids and oligonucleotides were transiently transfected using Lipofectamine 2000 (Invitrogen, Carlsbad, CA, US) to overexpress and/or silence target genes for gene intervention. The corresponding sequences are provided in Table S2 (Supporting Information).

### RNA Sequencing Assay

In the previous study, miR‐32533 was identified in APP/PS1 mice using high‐throughput sequencing.^[^ ^47^ ^]^ The raw data of miRNA profiles have been submitted to the Gene Expression Omnibus under dataset GSE194137. The miRDeep software (version 2.0.0.8) was used to predict novel sequence miRNAs.

### Adenovirus Vectors and Intracerebroventricular Injections

AAV‐loaded gene vectors were provided by OBIO Technology (Shanghai, China), including scrambled sequences, miR‐32533 mimics, miR‐32533 sponges, CREB5, and CREB5 shRNA. These vectors expressed a green fluorescent protein (GFP) with the following sequence structure: AAV‐CMV‐GFP‐scrambled sequences, AAV‐CMV‐GFP‐miR‐32533 mimics, AAV‐CMV‐GFP‐miR‐32533 sponges, AAV‐CMV‐GFP‐CREB5, and AAV‐CMV‐GFP‐CREB5 shRNA.

Six‐month‐old APP/PS1 mice were injected in the lateral ventricle with AAV‐scrambled sequences, AAV‐miR‐32533 mimics, AAV‐miR‐32533 mimics + AAV‐CREB5, AAV‐miR‐32533 sponges, or AAV‐miR‐32533 sponges + AAV‐CREB5 shRNA (a total of 50 mice divided into 5 groups). Age‐matched WT mice received intracerebroventricularly injections of AAV‐scrambled sequences, AAV‐miR‐32533 mimics, or AAV‐miR‐32533 mimics + AAV‐CREB5 (30 mice divided into 3 groups). Each group comprised five males and five females. Prior to intracerebroventricular injection, the viral titers of AAV‐scrambled sequences, AAV‐miR‐32533 mimics, AAV‐miR‐32533 sponges, AAV‐CREB5, and AAV‐CREB5 shRNA were determined using qPCR analysis as follows: 2.91 × 10^12^ V•g mL^−1^, 1.07 × 10^13^ V•g mL^−1^, 1.06 × 10^13^ V•g mL^−1^, 4.68 × 10^12^ V•g mL^−1^, and 1.96 × 10^12^ V•g mL^−1^. These viruses were diluted with saline to a standard concentration of 1.5 × 10^12^ V•g mL^−1^, and 2 µL of the diluted solution was injected into the mouse lateral ventricle using a brain stereotaxic apparatus (RWD Corp., Shenzhen, China) according to brain atlas^[^ ^48^ ^]^ as follows: medial–lateral, 1.0 mm; anterior–posterior, 0.5 mm; and dorsal–ventral, 3.0 mm.

### Morris Water Maze Test

The MWM test was used to assess the cognitive performance of APP/PS1 and WT mice genetically manipulated with AAV‐encapsulated miR‐32533 and CREB5 at 7 months. Briefly, the MWM test comprised five consecutive days of spatial navigation training, followed by probe trials conducted on days 6 and 7. Across these training days, the mice in each treatment group were instructed to locate a fixed platform from different entry points in four quadrants. The time required to successfully reach the platform (escape latency) was used as an indicator of spatial learning ability. In the probe trials, the duration spent in the quadrant of the removed platform and the frequency of crossings over the platform location were recorded as indicators of spatial memory.

### Human Blood Collection and Preparation

Peripheral blood samples were collected from 12 HAVs and 13 AD patients diagnosed at Xuanwu Hospital of Capital Medical University. This study was approved by the Human Ethics Committee of Xuanwu Hospital of Capital Medical University (approval number: linyanshen [2022]095). Informed consent was obtained from all participants. Detailed clinical information of the participants is shown in Table S3 (Supporting Information). The plasma was isolated by centrifugation and total RNA was extracted using a miRNeasy serum/plasma kit (Qiagen, Duesseldorf, Germany). The relative expression of miR‐32533 was determined as described in the “RNA isolation and qPCR analysis” section.

### RNA Fluorescence In Situ Hybridization (FISH)

The commercially available RNA FISH kit (Genepharma, Shanghai, China) was used to determine the distribution of miR‐32533 expression in the brain, following the manufacturer's direction. In brief, frozen coronal sections of WT and APP/PS1 mouse brain were equilibrated to room temperature before being digested with proteinase K at 37 °C for 20 min. As for mouse primary neurons, the cells were fixed with 4% paraformaldehyde for 15 min and then treated with 0.1% Triton X‐100 for 5 min. Afterward, both the sections and mouse primary neurons were immersed in a 1×blocking solution at 37 °C for 30 min and incubated in a preheated denaturing solution at 78 °C for 8 min. Subsequently, sections and primary neurons were incubated overnight at 37 °C with 1 µm SA‐Cy3 and a biotinylated miR‐32533 or 18S ribosomal RNA (rRNA) probe. DAPI staining was employed to identify the nuclei. Finally, fluorescent microscopy was used to visualize the miR‐32533 expression in the brain and cell samples. 18S rRNA was utilized as a positive control for the FISH procedure, ensuring the reliability of the hybridization signals (Figure S9, Supporting Information). The probes used for FISH analysis are shown in Table S4 (Supporting Information).

### Chromatin Immunoprecipitation (ChIP)

After APPswe cells were transfected with CREB5, the ChIP assay was conducted using the Pierce Magnetic ChIP Kit (Thermo Fisher Scientific, USA). The process involved cross‐linking cells with 1% formaldehyde, followed by lysis in a buffer solution containing Halt Protease and Phosphatase Inhibitor Cocktail. Then, nuclei were digested using MNase buffer, and chromatin was fragmented via ultrasound. The sheared chromatin was incubated with anti‐CREB5 antibody (A14635, ABclonal) or anti‐IgG at 4 °C overnight, followed by the addition of ChIP Grade Protein A/G Magnetic Beads for 2 h at 4 °C. The magnetic beads containing the eluted protein‐chromatin complex were separated using a magnetic force. The eluted and purified DNA products were subjected to qPCR to detect the enrichment of CREB5 bound to the promoter regions of genes responsible for encoding ADAM10, BACE1, and PS1. The primers used are shown in Table S4 (Supporting Information).

### Cell Viability

APPswe cells were transfected with the corresponding target gene (miR‐32533 mimics, miR‐32533 inhibitor, NC/NCI) for gene intervention. The cell viability was assessed using an MTS reagent (Promega, Madison, WI, USA), with absorbance measured at 490 nm using a Spark 20 m microplate reader (Tecan Group Ltd., Mannedorf, Switzerland).

### Measurement of Oxidative Stress Indicators

The level of ROS in genetically modified APPswe cells was determined by measuring the oxidation of DCFH‐DA to 2,7‐dichlorodihydrofluorescein using a ROS detection kit (Jiangsu AIDISHENG Biotechnology Co., Ltd., China) according to the manufacturer's instructions. Representative images were captured using an inverted fluorescence microscope, and the level of intracellular ROS was represented by the mean average fluorescent intensity using a 488/20 nm excitation and 525/20 nm emission filter. In the meantime, the levels of oxidative stress biomarkers, including SOD, GSH, and MDA, were accurately measured in APPswe cell extractions and in the cortex and hippocampus of APP/PS1 mice, utilizing commercial assay kits (Jiangsu AIDISHENG Biotechnology Co., Ltd., China) following the manufacturers' instructions.

### Apoptosis Assay

To examine the effect of miR‐32533 on apoptosis, a One‐step TUNEL in situ Apoptosis Kit (Pricella Biotechnology Co., Ltd., Wuhan, China) was used to perform the TUNEL assay. After gene modification, APPswe cells were immersed in 4% paraformaldehyde, followed by permeabilization with 0.2% Triton X‐100. The cells were equilibrated in TdT Equilibration Buffer for 30 min at 37 °C, then incubated with the labeling mixture for 4 h at 37 °C in the dark. Apoptotic cells were detected using fluorescence microscopy at a 488 nm excitation wavelength, followed by quantification analysis of apoptosis‐positive cells using ImageJ software (National Institutes of Health, Bethesda, MD, USA).

### RNA Isolation and qPCR Analysis

Total RNA was extracted from tissues and APPswe cells using Trizol reagent (Takara, Beijing, China). miRNA gene expression analysis was performed using the miRNA 1st Strand cDNA Synthesis Kit (by stem‐loop) and miRNA Universal SYBR qPCR Kit (Vazyme Biotech, Nanjing, China). Subsequently, mRNA transcription analysis was performed using the HiScript II cDNA Synthesis Kit and ChamQ SYBR qPCR Kit (Vazyme Biotech, Nanjing, China). The relative expression levels of mRNA and miRNA were calculated with the standardized 2^−ΔΔCT^ method, using GAPDH and U6 as endogenous controls, respectively. cDNA synthesis and PCR reactions were conducted on serially diluted miRNA standards to quantify miRNA levels in different tissues. A standard curve was generated by plotting the cycle threshold (Ct) values against the known concentrations of the miRNA standards, allowing for interpolation of miRNA concentrations based on Ct values obtained from the samples.^[^ ^49^, ^50^ ^]^ The primer sequences for qPCR are provided in Table S5 (Supporting Information).

### Western Blot

Western blot analysis was used to assess gene expression at the translational level according to previously described protocols.^[^ ^21^, ^22^, ^51^, ^52^ ^]^ In brief, proteins extracted from tissues and cells underwent a series of treatments, including quantification using bicinchoninic acid protein assay kit (Jiangsu Cowin Biotech Co., Ltd, Beijing, China), 10% sodium dodecyl sulfate‐polyacrylamide gel electrophoresis, transfer onto polyvinylidene fluoride membranes (Millipore, Massachusetts, USA), 5% nonfat milk blocking, incubation with primary and secondary antibodies, washing, and imaging. The corresponding antibodies used for Western blot analysis are detailed in Table S6 (Supporting Information).

### Northern Blot

Total RNA from WT mice was extracted using the PureLink miRNA Extraction Kit (Thermo Fisher Scientific, USA) and assayed using the Highly Sensitive MiRNA Northern Blotting Assay Kit (Signosis, Santa Clara, CA, USA) according to the manufacturer's instructions. Briefly, RNA samples were denatured and electrophoresed on polyacrylamide gels, transferred to nylon membranes, crosslinked under UV irradiation, hybridized with biotin‐labeled probes for 18 h at 42 °C, and finally detected through chemiluminescence imaging (Vilber Lourmat, Marne‐la‐Vall'ee, France). The biotin‐labeled probes were synthesized by Sangon Biotech Co., Ltd. (Shanghai, China), as detailed in Table S5 (Supporting Information). U6 snRNA was used as an endogenous control.

### miRNA Protein‐Coding Potential Analysis

The CPAT platform (https://wlcb.oit.uci.edu/cpat/index.php) was used to predict the protein‐coding potential of miR‐32533. To experimentally validate this potential, the pre‐miR‐32533 sequence was cloned upstream of the p3×FLAG vector using a HindIII/EcoRI restriction site, synthesized by Beijing Lainuo Biotechnology Co., Ltd. (Beijing, China). HEK293 cells were transfected with 2 µg of CMV‐pre‐miR‐32533‐3×FLAG plasmid or p3×FLAG vector using Lipofectamine 2000. Western blot analysis was conducted to detect the expression of FLAG Tag (Cell Signaling Technology, Cat# 8146S).

### ELISA Analyses

Levels of Aβ_1‐40_, Aβ_1‐42_, sAPPβ, sAPPα (Shanghai Enzyme‐linked Biotechnology Co., Ltd., China), TNF‐α, and IL‐6 (Biolegend, California, USA) were measured in APPswe cells and/or brain tissues of APP/PS1 mice, as well as levels of Aβ_1‐40_, Aβ_1‐42_, and p‐Tau217 (Shanghai Enzyme‐linked Biotechnology Co., Ltd., China) in the plasma of AD patients and HAVs, using commercially available ELISA kits according to the manufacturers' instructions.

### Clinical Potential Analyses of miR‐32533

The Pearson correlation coefficient was employed to assess the linear relevancy between miR‐32533 and MMSE scores, Aβ_1‐42_/Aβ_1‐40_ ratio, and p‐Tau217 level of AD patients. The ROC curve was plotted to analyze its diagnostic sensitivity and specificity.

### Luciferase Activity Assay

A 300 bp CREB5 sequence covering the miR‐32533 binding sites upstream and downstream was ligated into the luciferase vector pmirGLO to obtain the reporter gene (pGL‐3′UTR‐CREB5‐Wt), and the binding site was entirely mutated to get the reporter gene (pGL‐3′UTR‐CREB5‐Mut). These reporter genes were co‐transfected with miR‐32533 or NC into HEK293 cells. The potential binding sequences for ADAM10, BACE1, and PS1 with CREB5 were integrated into the promoter regions of the pGL3‐Basic vectors, along with their respective mutant counterparts. These recombinational pGL3‐Basic plasmids and Renilla (an endogenous control plasmid) were co‐transfected with the CREB5 overexpression plasmid into APPswe cells. All plasmids were synthesized by Sangon Biotech.

Luciferase activity and Renilla fluorescence were assessed using a commercial dual luciferase activity detection kit (Vazyme, Nanjing, China) and a microplate reader (Tecan Group Ltd.), with the results normalized to Renilla luminescence.

### Histochemical Analysis

Four mice per group were anesthetized with isoflurane. The brain tissue was extracted, immersed in 4% paraformaldehyde, and dissected into 20 µm frozen sections. Nissl vesicle and Aβ burden were measured in the brains of APP/PS1 mice post‐genetic intervention with AAV‐loaded scrambled sequences, miR‐32533 mimics, miR‐32533 sponges, CREB5, or CREB5 shRNA. Toluidine blue (Servicebio, Wuhan, China), an alkaline dye, stains Nissl bodies purple‐blue to assess the physiological state of neurons. Aβ deposition was detected using an Aβ (6E10) primary antibody (Biolegend, San Diego, CA, USA) and then enhanced horseradish peroxidase‐diaminobenzidine substrate chromogenic kit (Zhongshan Jinqiao Biotechnology Co., Ltd., Beijing, China). Nissl bodies and Aβ (6E10)‐positive plaques within the hippocampus and cortex were captured utilizing a Panoramic MIDI Digital Slide Scanner (3DHISTECH, Budapest, Hungary). Quantitative analyses of Nissl bodies and Aβ (6E10)‐positive signals and numbers were conducted using ImageJ software.

### Bioinformatics Analysis

The secondary structure of precursor miR‐32533 was predicted using online RNAfold software (http://rna.tbi.univie.ac.at/cgi‐bin/RNAWebSuite/RNAfold.cgi). Homologous sequence analysis was performed using the UCSC gene database and Jalview Software. The phylogenetic tree of miR‐32533 was described using the Clustal Omega (http://www.clustal.org/) and miRBase (http://www.mirbase.org/) platforms. Target prediction for miR‐32533 was conducted using the miRDB platform (https://mirdb.org/). Kyoto Encyclopedia of Genes and Genomes (KEGG) pathway enrichment of miR‐32533 targets was performed using the KEGG mapper online platform (https://www.kegg.jp/kegg/mapper/).

### Statistical Analysis

Statistical analysis was performed using GraphPad Prism 8.0 (GraphPad Inc., La Jolla, CA, USA) and SPSS 25.0 software (IBM, Armonk, NY, USA). Data normality was assessed using the Shapiro–Wilk normality test. A two‐tailed Student's *t*‐test was utilized for statistical comparisons involving two independent groups. For evaluating statistical significance among multiple groups, one‐way ANOVAwas employed, followed by Tukey's *post hoc* test. To analyze the expression of miR‐32533 and CREB5 in APP/PS1 and 5×FAD mice of different ages, two‐way ANOVA and Sidak's multiple comparisons test were performed. Repeated measures ANOVA was used to compare cognitive ability in the MWM test, including escape latency and swimming speed between APP/PS1 and WT mice. For molecular biological experiments, each group consisted of 3–4 replicates. In the MWM test, each experimental group comprised ten mice. Regarding experiments with human samples, the study included 12 HAVs and 13 AD patients. The results are presented as mean ± SD. Any result with a *p*‐value less than 0.05 is considered to indicate a statistically significant difference.

## Conflict of Interest

The authors declare no conflict of interest.

## Author Contributions

R.L. and Z.L. conceived and directed the projects. R.L. obtained the funding. R.L., L.Z., and Z.C. wrote the manuscript. L.Z., Z.C., J.L., K.Z., F.L., and T.S. conducted experiment execution, data collection, and data analysis. L.Z., Z.C., and J.L. contributed equally to this work. All authors read and approved the final manuscript.

## Supporting information



Supporting Information

## Data Availability

The data that support the findings of this study are available from the corresponding author upon reasonable request.
