# Supplementary material for: miR‐32533 Reduces Cognitive Impairment and Amyloid‐β Overload by Targeting CREB5‐Mediated Signaling Pathways in Alzheimer's Disease
Source: Adv Sci (Weinh). 2025 Jan 22;12(10):2409986. doi: 10.1002/advs.202409986 (PMC11905094; doi:10.1002/advs.202409986)
Supplement: Supplementary file 1 — Supporting Information [file ADVS-12-2409986-s001.docx]

Supporting Information

**miR-32533 Reduces Cognitive Impairment and Amyloid-β Overload by Targeting CREB5-Mediated Signaling Pathways in Alzheimer’s Disease**

*Li Zeng,^1,2#^ Zhongdi Cai,^1,2#^ Jianghong Liu,^3#^ Kaiyue Zhao,^1,2^ Furu Liang,^4^ Ting Sun,^1,2^ Zhuorong Li,^1,2*^ Rui Liu^1,2*^*

**Table S1.** Potential interaction sites between CREB5 and the promoter regions of BACE1, PS1, and ADAM10 identified using the JASPAR software

| **Target** | **Target promoter sequence ID** | **Start** | **End** | **Predicted sequence** | **Score** |
| --- | --- | --- | --- | --- | --- |
| BACE1 | NC_000011.10:c117318256-117316157 | 899 | 910 | CAACATGGTGAA | 10.313501 |
|  |  | 341 | 353 | TTTTGAGTCAATC | 7.279993 |
|  |  | 983 | 995 | GGCTGAGGCATGA | 6.8994513 |
| PS1 | NC_000014.9:73134417-73136318 | 390 | 402 | GCGTGAGCCACCG | 6.990054 |
|  |  | 1644 | 1656 | TTTTGATTCATTG | 6.8208747 |
|  |  | 1026 | 1040 | TGGTGTGATCTCAGC | 6.5495834 |
| ADAM10 | NC_000015.10:c58751707-58749608 | 1168 | 1180 | AAATGAGTTACGA | 6.487509 |
|  |  | 206 | 220 | TTTGATGATTCCATG | 5.7803617 |
|  |  | 109 | 123 | ACAGGTGATATGAGA | 3.2491658 |

**Table S2.** Nucleotide sequence used for transfection

| **Name** | **RNA Sequence** |
| --- | --- |
| negative control (NC) | sense: 5'-UUCUCCGAACGUGUCACGUTT-3' |
|  | antisense: 5’-ACGUGACACGUUCGGAGAATT-3’ |
| miR-32533 mimics | sense: 5’-UCUCUUCUGCUCUGUGUCACAGC-3’ |
|  | antisense:5’-UGUGACACAGAGCAGAAGAGAUU-3’ |
| negative control inhibitor (NCI) | 5’- CAGUACUUUUGUGUAGUACAA -3’ |
| miR-32533 inhibitor | 5’- GCUGUGACACAGAGCAGAAGAGA-3’ |
| CREB5 siRNA | sense: 5’-GCAACAAGUCAUCCAGCAUAATT -3’ |
|  | antisense: 5’-UUAUGCUGGAUGACUUGUUGCTT -3’ |

**Table S3.** Clinical data from AD patients and healthy age-matched volunteers

| **Variables** | **HAVs** | **AD** | ***P*-value** |
| --- | --- | --- | --- |
| Number of subjects | 12 | 13 | / |
| Gender (male/female) | 7/5 | 5/8 | / |
| Age (years, mean ± SD) | 68.9 ± 8.33 | 74.5 ± 8.95 | 0.12 |
| MMSE (score, mean ± SD) | 27± 1.41 | 17.8 ± 8.05 | <0.001 |

Note: AD, AD patients; HAVs, healthy age-matched volunteers; MMSE, mini-mental status examination.

**Table S4.** Primers used for ChIP-qPCR assay and probes for FISH analysis

| **Primer Name** | **Primer Sequence (5’-3’)** |
| --- | --- |
| PS1-1-F | GCGATAGGAATGTGAAGGGATA |
| PS1-1-R | CAGGAGAATCGCTTGAACCCGTGAG |
| PS1-2-F | TTTCTCAGGTTCTCGCCACC |
| PS1-2-R | CGAACCAGCTGCATCACAAT |
| PS1-3-F | ACGGGGTTTCTCCATGTTG |
| PS1-3-R | AATAGGCAGGGTGCGGTGGC |
| ADAM10-1-F | AAATGGCCATAAAAAGTTCTAGTTC |
| ADAM10-1-R | ACATCTTCTCCCCAACTTCT |
| ADAM10-2-F | TCATTTGTTGACAGCACTAC |
| ADAM10-2-R | GGAAATAGTTACCTGCAGAC |
| ADAM10-3-F | CATTCGTGAGATTCTGCCAT |
| ADAM10-3-R | GCTGCAACATTAATAGGCTC |
| BACE1-1-F | ATTAGCTGGGTATGGTGGCG |
| BACE1-1-R | GGCATGGTCTCAAGTCTGCA |
| BACE1-2-F | GCAACTAATGATTCCTTTGCTTTCC |
| BACE1-2-R | GCCAAGATGGTCTCGATCTCC |
| BACE1-3-F | ACAGTGGTCCATGCCTATAATCC |
| BACE1-3-R | AAGTCTGCAACCTCCGCCTC |
| miR-32533 probe | GCTGTGACACAGAGCAGAAGAGA |
| 18S probe | CTGCCTTCCTTGGATGTGGTAGCCGTTTC |

Note: F, forward primer; R, reverse primer.

ADAM10-1 (located at chr15:c58,750,540-58,750,528), ADAM10-2 (located at chr15:c58,751,599-58,751,585), ADAM10-3 (located at chr15: c58,751,505-58,751,488); BACE1-1 (located at chr11:c117,317,274-117,317,262), BACE1-2 (located at chr11:c117,317,916-117,317,904), BACE1-3 (located at ch11:c117,317,358-117,317,347); PS1-1 (located at chr14:731,354,42-73,135,456), PS1-2 (located at chr14:73,136,060-73,136,072), PS1-3 (located at chr14:73,134,806-73,134,818).

**Table S5.** Primers used for qPCR assay and probes employed for Northern blot

| **Primer Name** | **Primer Sequence (5’-3’)** |
| --- | --- |
| miR-32533-RT | GTCGTATCCAGTGCAGGGTCCGAGGTATTCGCACTGGATACGACGCTGTG |
| miR-32533-F | CGCGTCTCTTCTGCTCTGTGT |
| miR-32533-R | AGTGCAGGGTCCGAGGTATT |
| U6-RT | GTCGTATCCAGTGCAGGGTCCGAGGTATTCGCACTGGATACGACAAAATA |
| U6-F | CAAATTCGTGAAGCGTTCCA |
| U6-R | AGTGCAGGGTCCGAGGTATT |
| CREB5-F | ATTGACTCACCACCCTGCTG |
| CREB5-R | GCATGAAGGTGGGAATGGGA |
| GAPDH-F | CAAATTCCATGGCACCGTCA |
| GAPDH-R | AGCATCGCCCCACTTGATTT |
| Creb5-F | CTCCACACTTGTTTGATTTATGAGG |
| Creb5-R | GTCATCTCGTGCTTGTGCCTA |
| Gapdh-F | TGTGTCCGTCGTGGATCTGA |
| Gapdh-R | CCTGCTTCACCACCTTCTTGA |
| miR-32533 (Northern blot) | GCTGTGACACAGAGCAGAAGAGA |
| U6  (Northern blot) | CACGAATTTGCGTGTCATCCT |

Note: F, forward primer; R, reverse primer; RT, reverse transcription primer.

**Table S6.** Primary and secondary antibodies used in Western blot analysis

| **Primary antibody** | **Dilution** | **Source** | **Secondary antibody** |
| --- | --- | --- | --- |
| Anti-tau (phospho S404) rabbit mAb | 1:1000 | Abcam | HRP-conjugated goat anti-rabbit or anti-mouse IgG (H+L) (1:5000, Gene-Protein Link) |
| Anti-tau (phospho S396) rabbit mAb | 1:1000 | Abcam |  |
| Anti-Tau rabbit mAb | 1:1000 | Abcam |  |
| Anti-beta Amyloid 1-42 rabbit pAb | 1:1000 | Abcam |  |
| Anti-ADAM10 rabbit pAb | 1:1000 | Abcam |  |
| Anti-Aβ (6E10) mouse mAb | 1:1000 | Biolegend |  |
| Anti-BACE1 rabbit mAb | 1:1000 | Abcam |  |
| Anti-Amyloid Precursor protein rabbit mAb | 1:1000 | Abcam |  |
| Anti-Presenilin/PS1 rabbit mAb | 1:1000 | Abcam |  |
| Anti-Bcl-2 rabbit mAb | 1:1000 | CST |  |
| Anti-Bax rabbit mAb | 1:1000 | Abcam |  |
| Anti-CREB5 rabbit pAb | 1:1000 | Immunoway |  |
| Anti-PARP rabbit pAb | 1:1000 | CST |  |
| Anti-Clevead PARP rabbit pAb | 1:1000 | CST |  |
| Anti-Caspase-3 rabbit pAb | 1:1000 | CST |  |
| Anti-Clevead Caspase-3 rabbit pAb | 1:1000 | CST |  |
| Anti-Cytochrome *c* rabbit pAb | 1:1000 | Abcam |  |
| Anti-GAPDH rabbit mAb | 1:1000 | Proteintech |  |

Note: Abcam, Cambridge, MA, USA; CST, Cell Signaling Technology, Danvers, MA, USA; Biolegend, San Diego, CA, USA; Proteintech, Chicago, IL, USA; Immunoway, Plano, TX, USA.


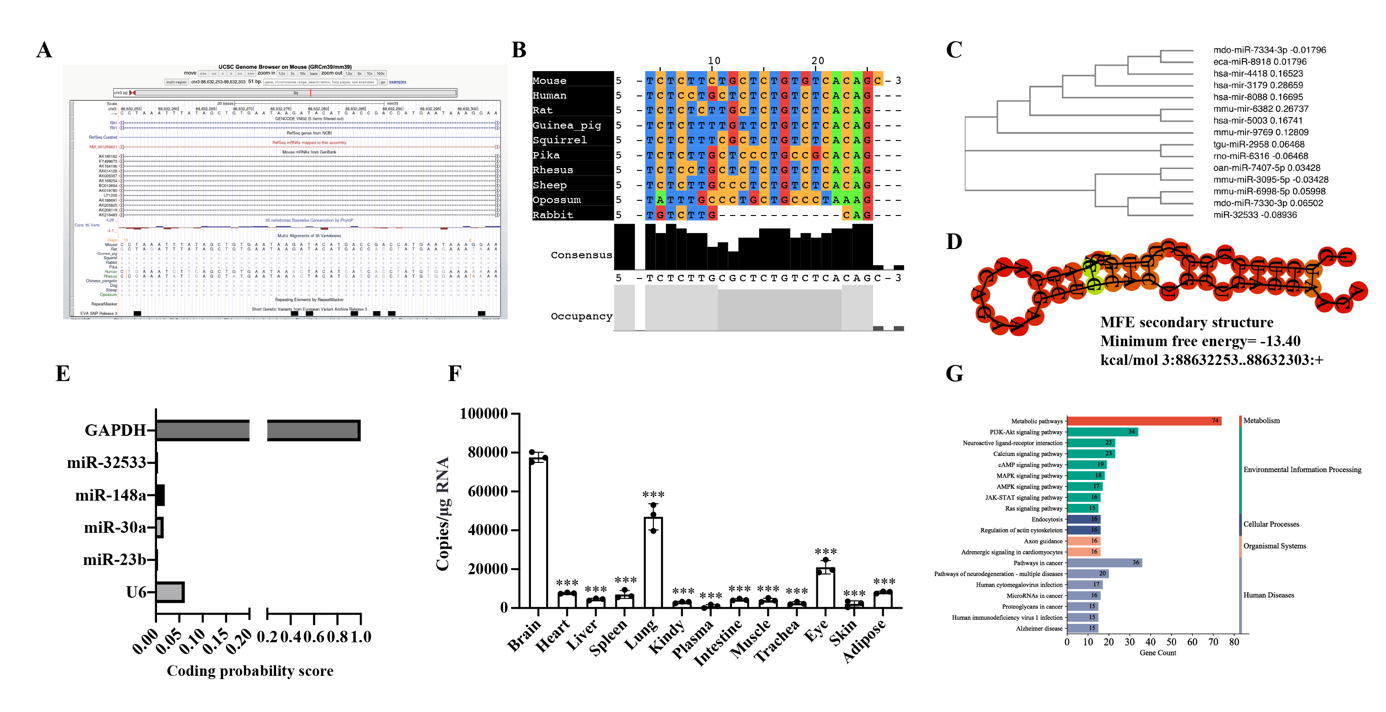


**Figure S1.** Gene annotation, sequence analysis, and functional prediction of miR-32533. A) The gene locus (chr3:88,632,253-88,632,303: +) of precursor miR-32533 sourced from the UCSC database. B) Sequence homology analysis of miR-32533 using the UCSC database and Jalview software. C) Construction of a phylogenetic tree displaying the genetic relationship between miR-32533 and known miRNAs using the miRBase database and the Clustal Omega platform. Local alignment methods in the EBI online platform (https://wwwdev.ebi.ac.uk/Tools/jdispatcher) were employed to calculate similarity. D) Secondary structure of miR-32533, predicted using RNAfold software, with a minimum free energy of -13.40 kcal/mol. E) Coding potential analysis for miR-32533 using CPAT platform. F) Relative miR-32533 copy number in thirteen tissues of WT mice detected using qPCR (*n*=3). Multi-group comparisons were performed using one-way ANOVA followed by Tukey’s *post* *hoc* test. Results are presented as mean ± SD. ^***^*p* < 0.001 versus Brain. G) Top 20 KEGG enrichment pathways for miR-32533-targeted genes using the KEGG mapper online platform (https://www.kegg.jp/kegg/mapper/).





**Figure S2.** Transfection efficiency *in vitro*. A) Transfection efficiency of negative control (NC) and miR-32533 mimics in APPswe cells (*n*=4). B) Transfection efficiency of negative control of inhibitor (NCI) and miR-32533 inhibitor in APPswe cells (*n*=4). Comparisons between the two groups were conducted using the two-tailed Student's *t*-test. Results are presented as mean ± SD. ^**^*p* < 0.05, ^***^*p* < 0.001 versus NC/NCI.





**Figure S3.** Role of miR-32533 in Tau protein phosphorylation *in vitro*. A) miR-32533 levels in SH-SY5Y cells post-transfection with pcDNA3.1-Tau or vector (*n*=3). B and C) Representative Western blot images B) and quantifications C) of pTS396, pTS404, Tau, and GAPDH in the Tau overexpressing SH-SY5Y cells transfected with miR-32533 mimics, inhibitor, or NC/NCI (*n*=3). Two-group comparisons in panel A were performed using the two-tailed Student's *t*-test. Multi-group comparisons in Panel C were performed using one-way ANOVA followed by Tukey’s *post* *hoc* test. Results are presented as mean ± SD.





**Figure S4.** ChIP-qPCR analysis of negative binding sites of CREB5 to promoter fragments of ADAM10, BACE1, and PS1. A) ChIP-qPCR analysis of anti-CREB5 or IgG-immunoprecipitated promoter fragments of ADAM10-1 (located at chr15:c58,750,540-58,750,528) and ADAM10-2 (located at chr15:c58,751,599-58,751,585), extracted from APPswe cells transfected with CREB5 (*n*=3). B) ChIP-qPCR analysis of anti-CREB5 or IgG-immunoprecipitated promoter fragments of BACE1-1 (located at chr11:c117,317,274-117,317,262) and BACE1-2 (located at chr11:c117,317,916-117,317,904), extracted from APPswe cells transfected with CREB5 (*n*=3). C) ChIP-qPCR analysis of anti-CREB5 or IgG-immunoprecipitated promoter fragments of PS1-1 (located at chr14:731,354,42-73,135,456) and PS1-2 (located at chr14:73,136,060-73,136,072), extracted from APPswe cells transfected with CREB5 (*n*=3). Multiple group comparisons were conducted using one-way ANOVA, followed by Tukey’s *post hoc* test for inter-group difference analysis. Results are presented as mean ± SD.





**Figure S5.** Transfection efficiency *in vitro*. A) Transfection efficiency of NC and CREB5 in APPswe cells (*n*=4). B) Transfection efficiency of NCI and CREB5 siRNA in APPswe cells (*n*=3). Two-group comparisons were performed using the two-tailed Student's *t*-test. Results are presented as mean ± SD. ^*^*p* < 0.05, ^***^*p* <0.001 versus NC/NCI.





**Figure S6.** Transfection efficiency of miR-32533 and CREB5 *in vivo*. A) Transfection efficiency of miR-32533 mimics in the brains of APP/PS1 mice using qPCR (*n*=4). B) Transfection efficiency of CREB5 in the brains of APP/PS1 mice using qPCR (*n*=4). C) GFP tag-based transfection efficiency of AAV-miR-32533 and AAV-CREB5 in the brains of APP/PS1 mice. Two-group comparisons were conducted using the two-tailed Student's *t*-test. Results are presented as mean ± SD. ^**^*p* < 0.01, ^***^*p* < 0.001 versus APP/PS1 mice scrambled control.


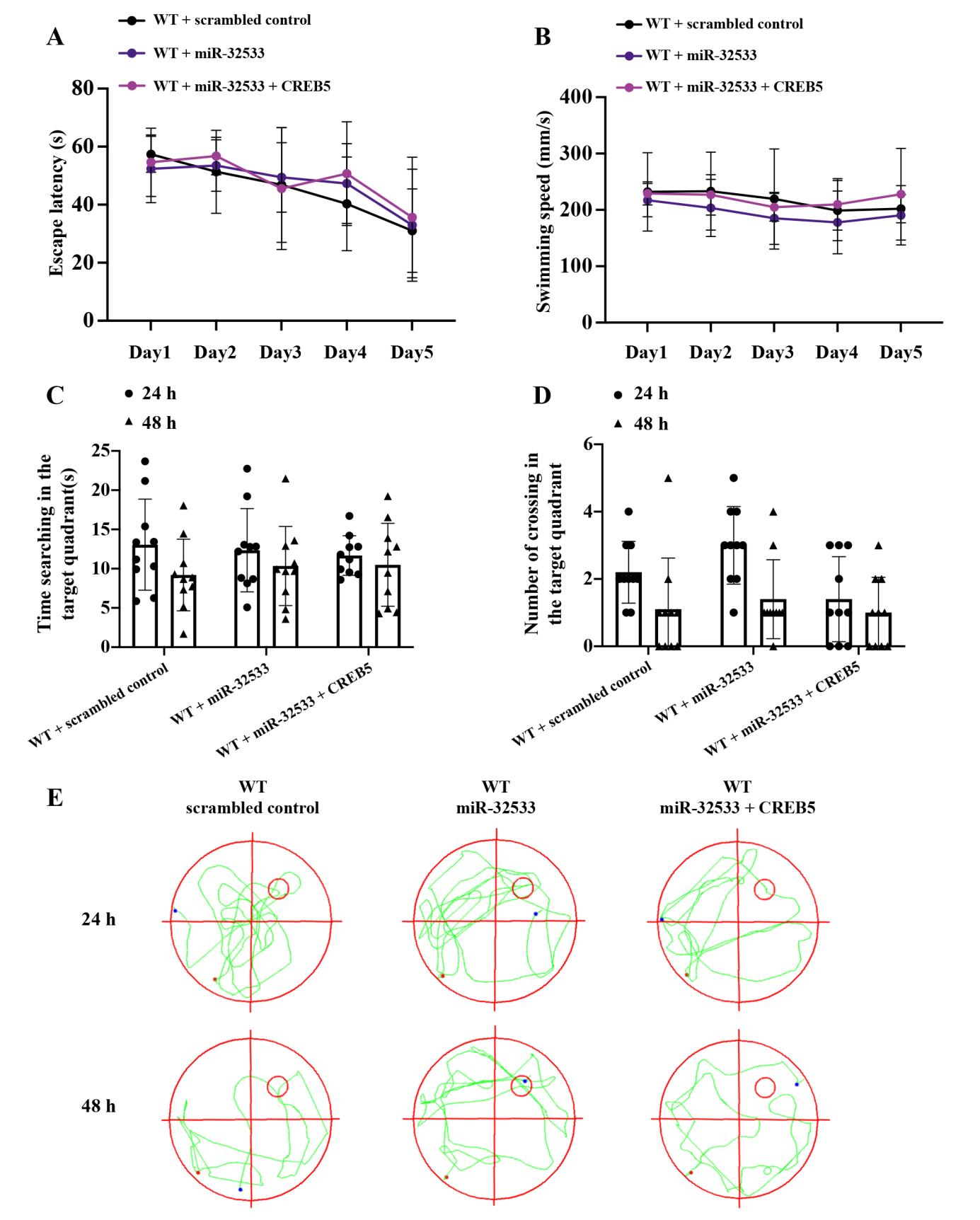


**Figure S7.** Effects of miR-32533 mimics alone or combined with CREB5 infusion on cognitive behavior in WT mice. A) Escape latency of WT mice in each treatment group over five days of navigation training in the MWM test. B) Swimming speed of WT mice during the navigation test in each treatment group. C) Time spent in the target quadrant on days 6 and 7 for each treatment group. D) Number of crossings through the target quadrant on days 6 and 7. E) Representative locomotor trajectories of WT mice at 24 h and 48 h of the post-training probe. Repeated measures ANOVA was applied for panels A and B, whereas one-way ANOVA was utilized for panels C and D, followed by Tukey's *post hoc* test for inter-group difference analysis. Results are presented as mean ± SD. *n*=10.





**Figure S8.** Transfection efficiency of miR-32533 sponges and CREB5 shRNA *in vivo*. A) Transfection efficiency of miR-32533 sponges in the brains of APP/PS1 mice using qPCR (*n*=4). B) Silencing efficiency of CREB5 shRNA in the brains of APP/PS1 mice using qPCR (*n*=4). C) GFP tag-based transfection efficiency of AAV-miR-32533 sponges and AAV-CREB5 shRNA in the brain of APP/PS1 mice. Two-group comparisons were performed using the two-tailed Student's *t*-test. Results are presented as mean ± SD. ^***^*p* < 0.001 versus APP/PS1 mice scrambled control.





**Figure S9.** Level of 18S *in vivo* and *in vitro.* A) Representative images of 18S levels in the brains of WT and APP/PS1 mice using FISH assay. Scale bar: 20 μm, 50 μm, 100 μm, or 1000 μm. B) Representative images of 18S levels in primary mouse hippocampal and cortical neuronal cells using FISH assay. Scale bar: 100 μm.


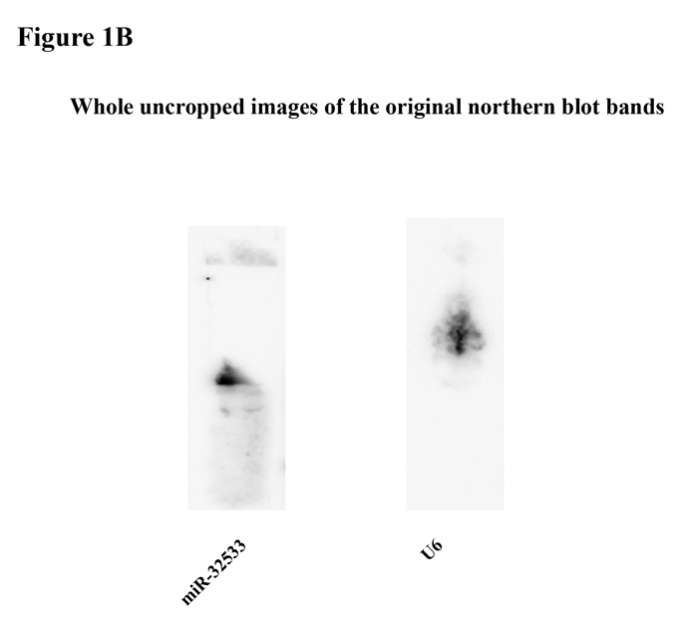


**Figure S10.** Whole uncropped images of the original blots of Figure 1B.





**Figure S11.** Whole uncropped images of the original blots of Figure 1D.


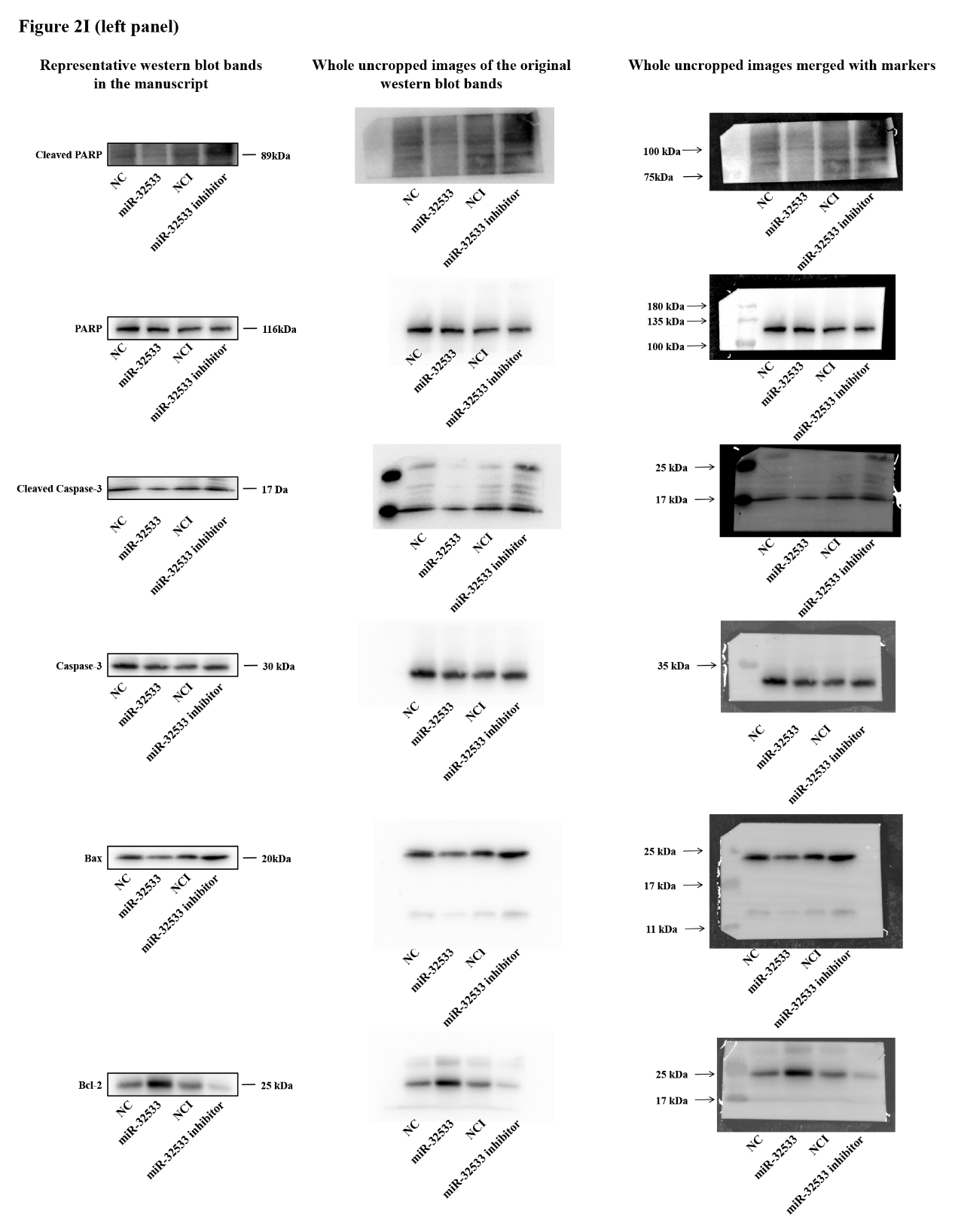


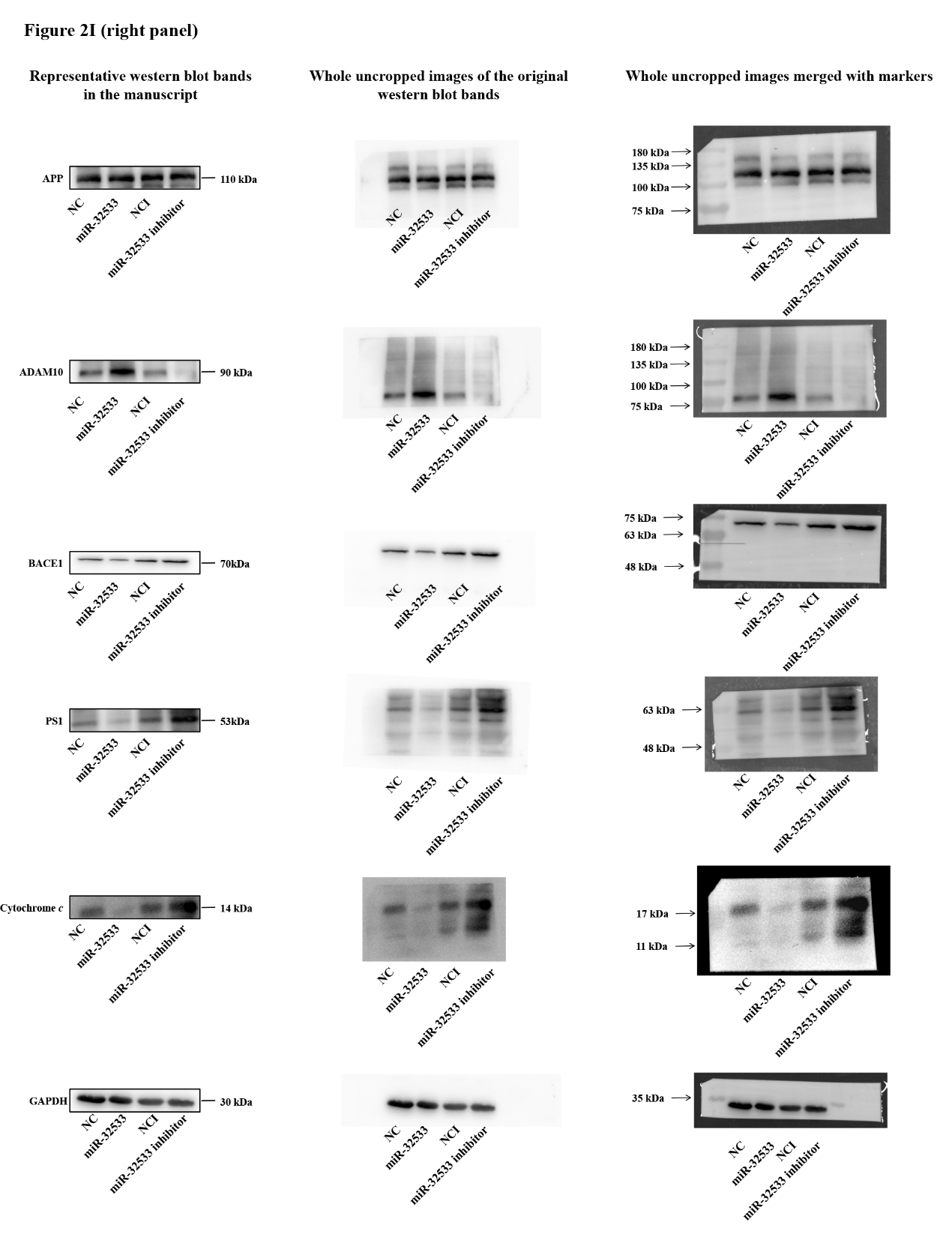


**Figure S12.** Whole uncropped images of the original blots of Figure 2I.


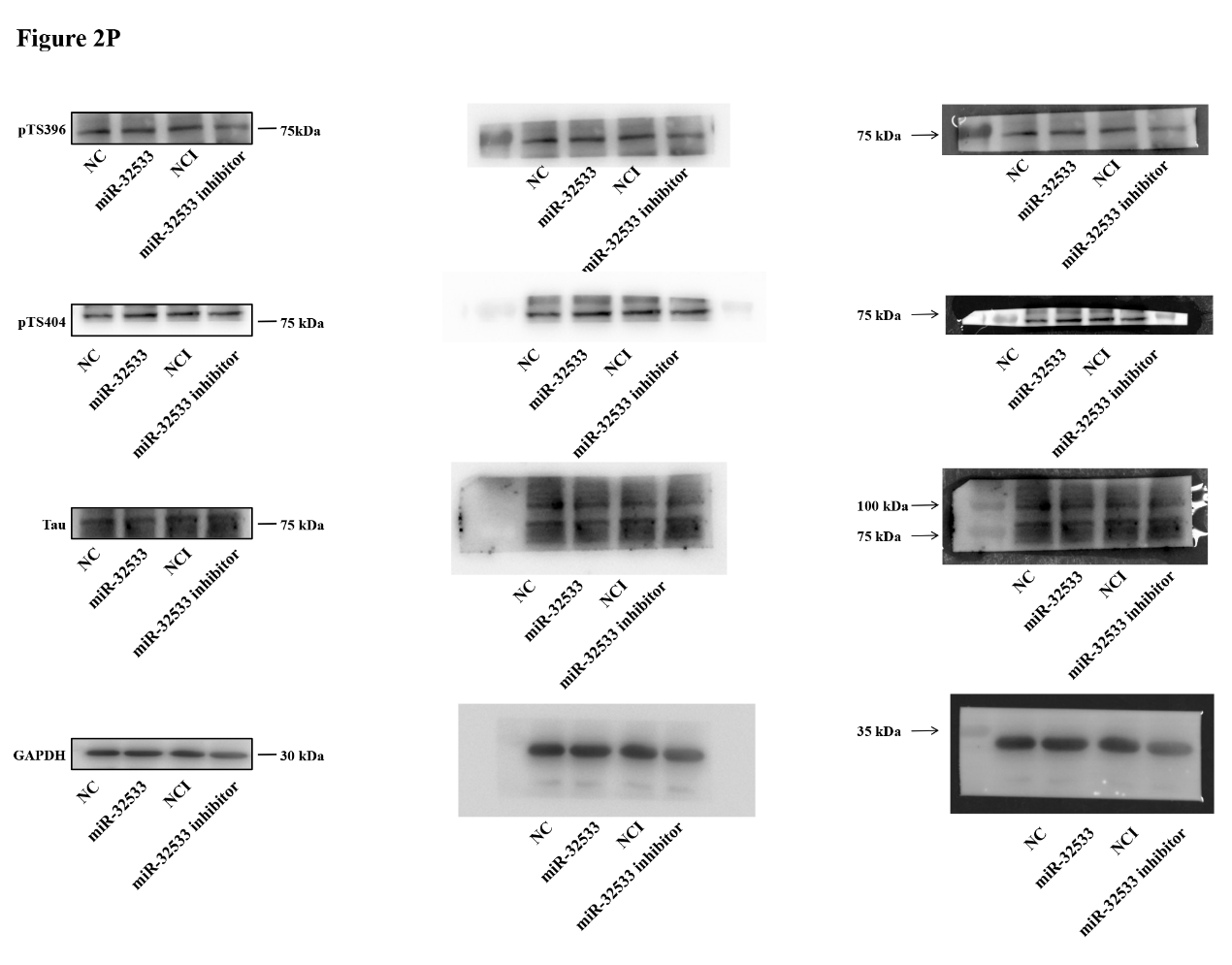


**Figure S13.** Whole uncropped images of the original blots of Figure 2P.


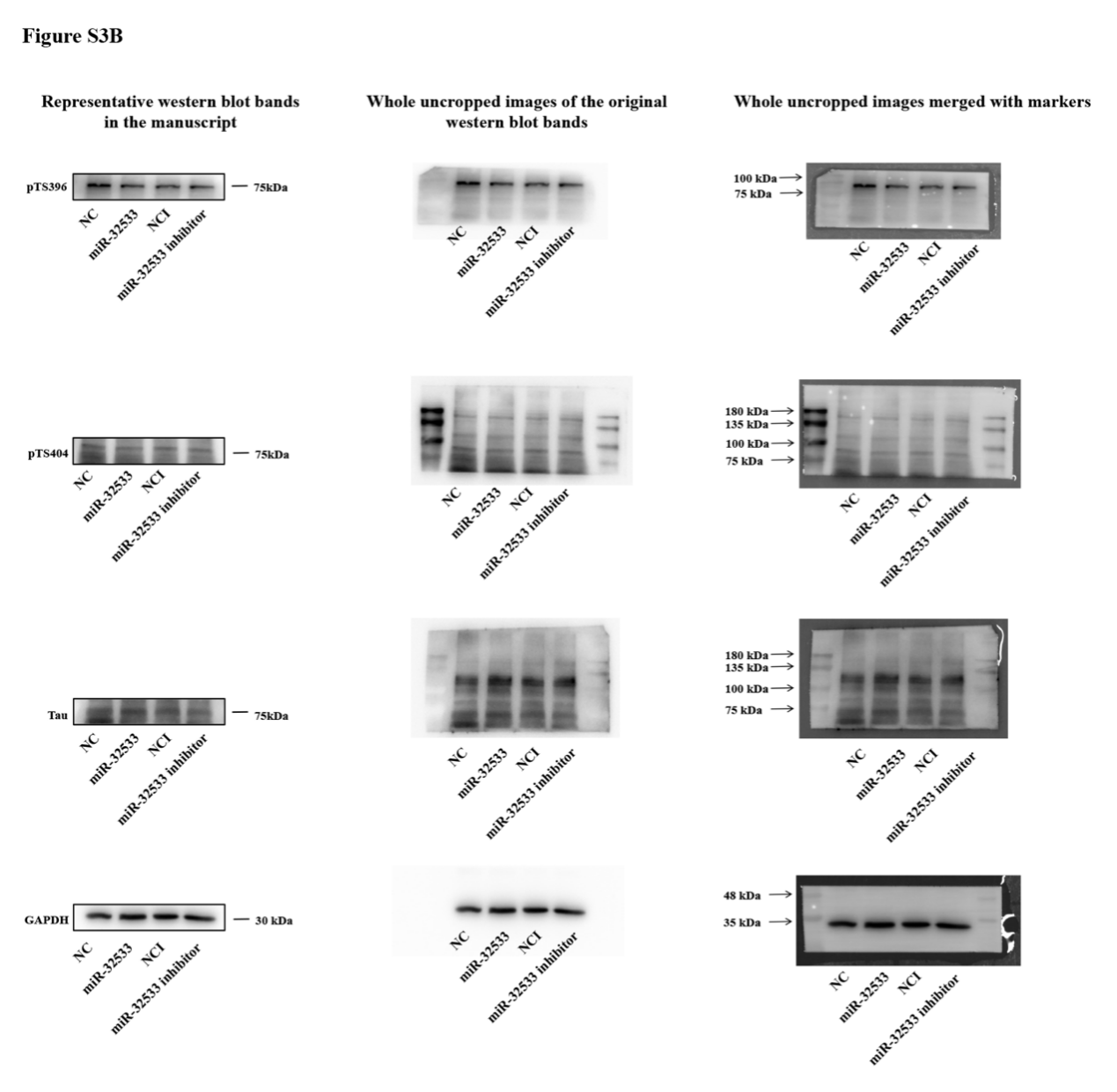


**Figure S14.** Whole uncropped images of the original blots of Figure S3B.


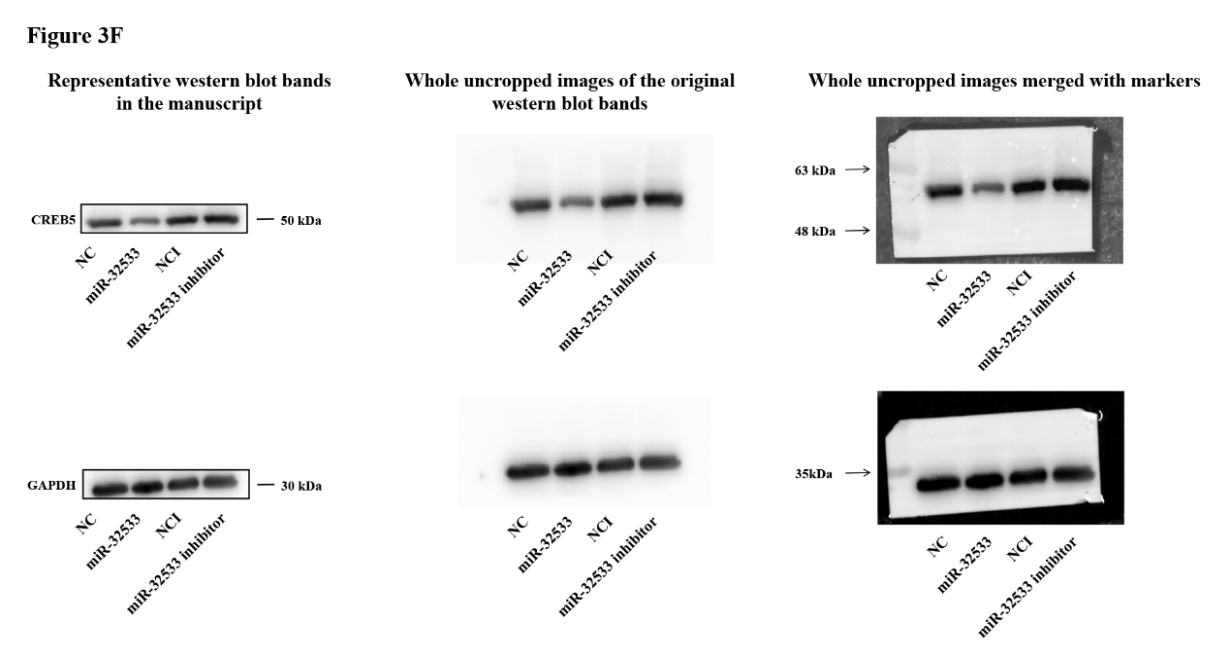


**Figure S15.** Whole uncropped images of the original blots of Figure 3F.


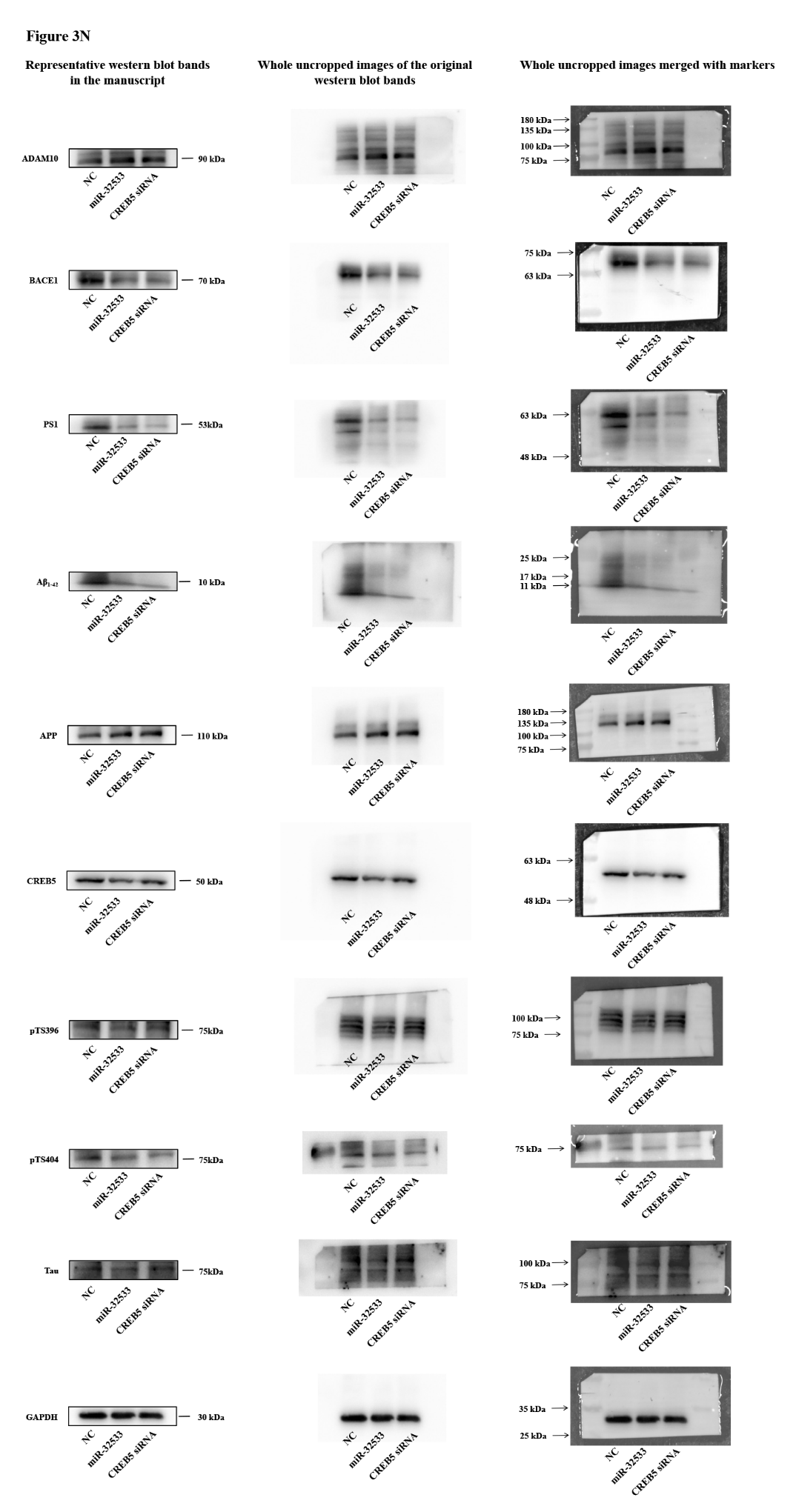


**Figure S16.** Whole uncropped images of the original blots of Figure 3N.


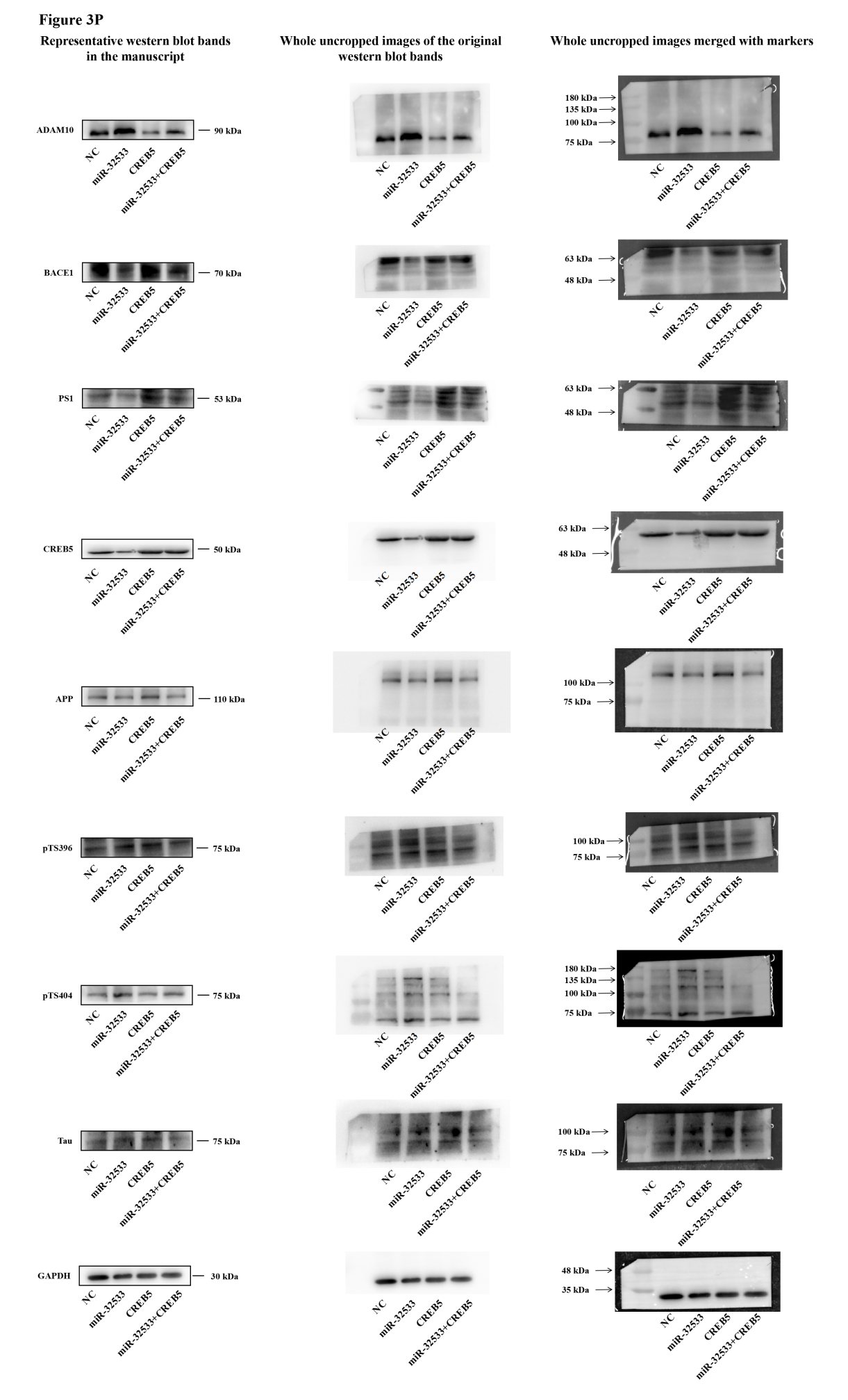


**Figure S17.** Whole uncropped images of the original blots of Figure 3P.


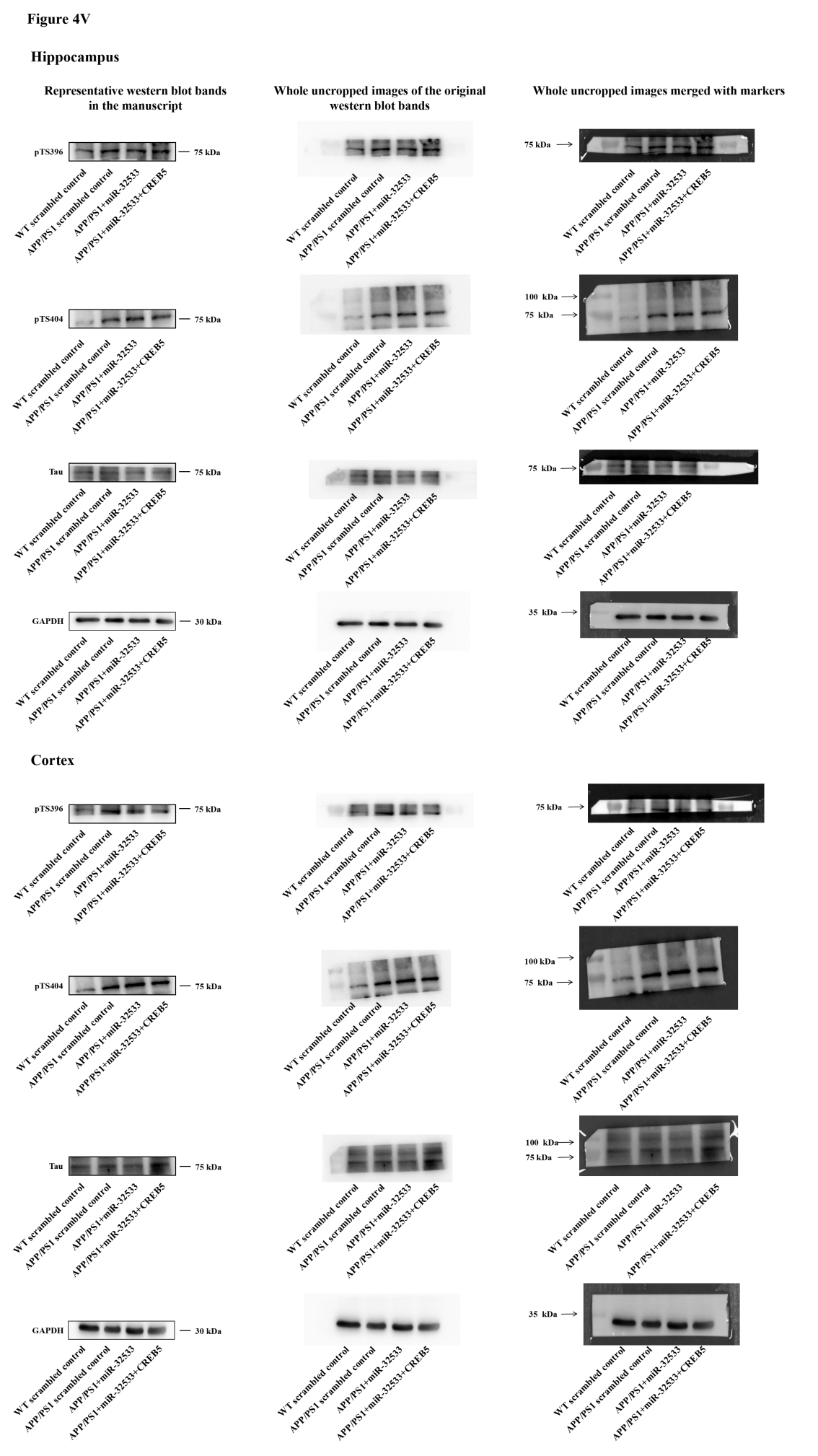


**Figure S18.** Whole uncropped images of the original blots of Figure 4V.


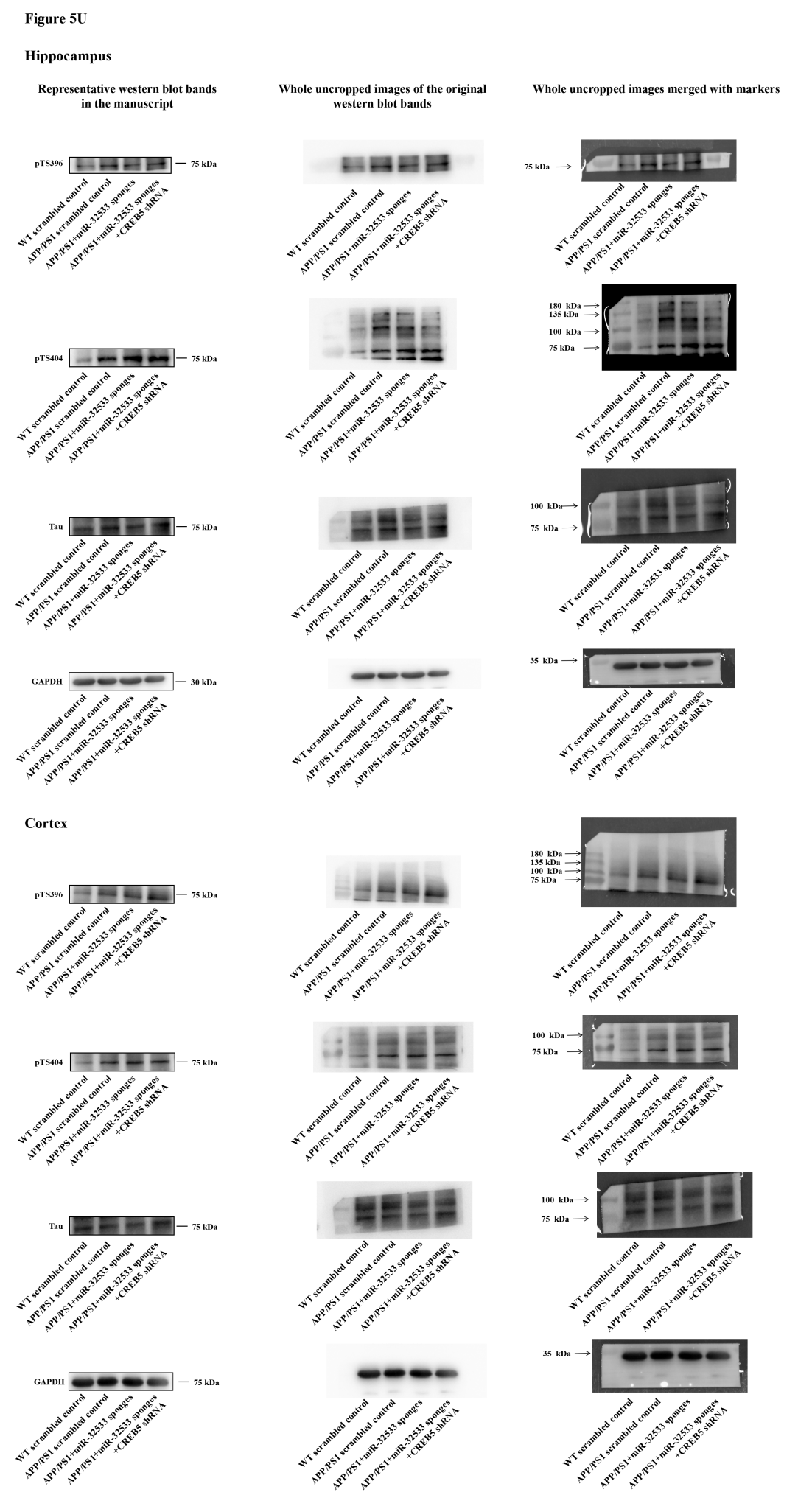


**Figure S19.** Whole uncropped images of the original blots of Figure 5U.




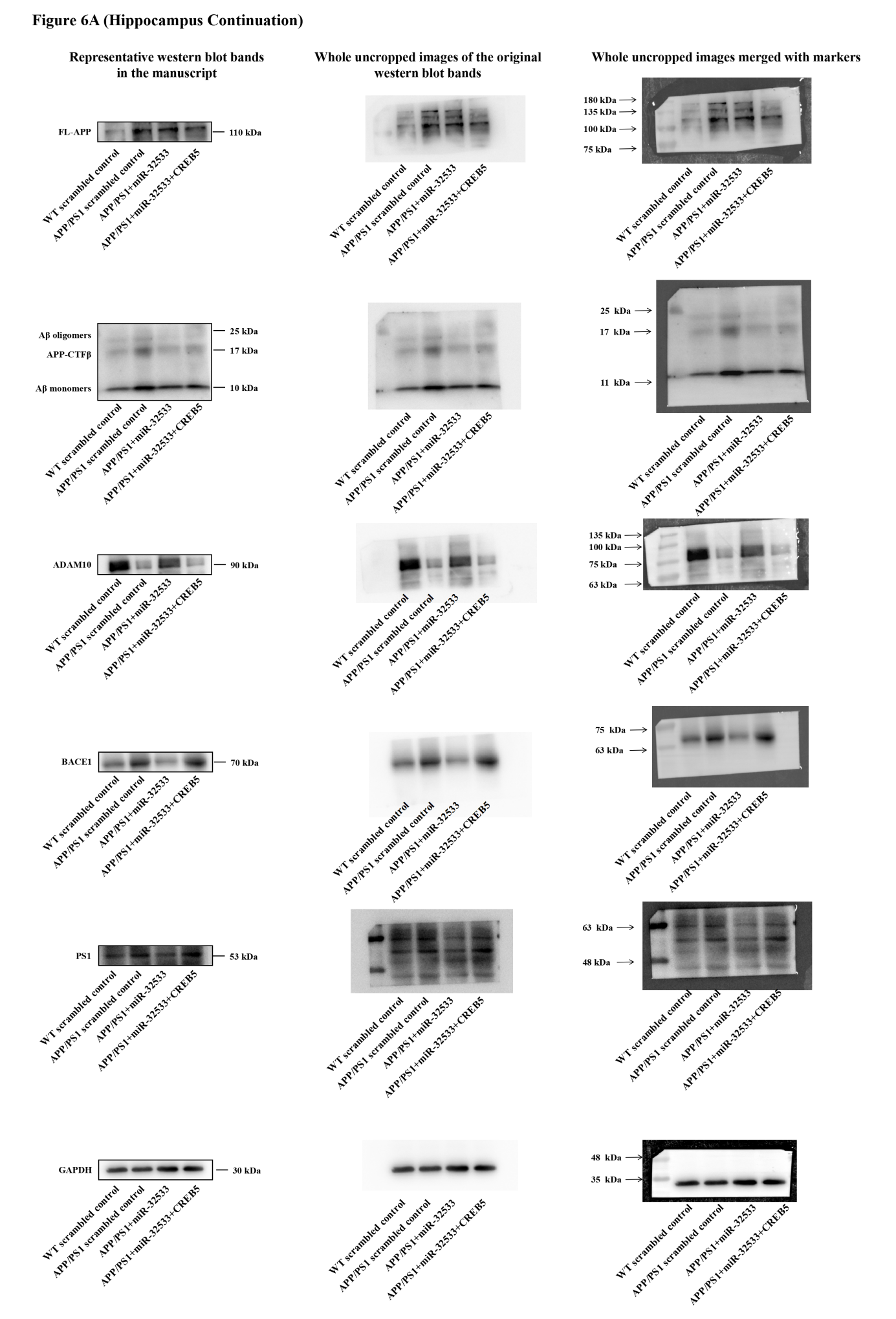

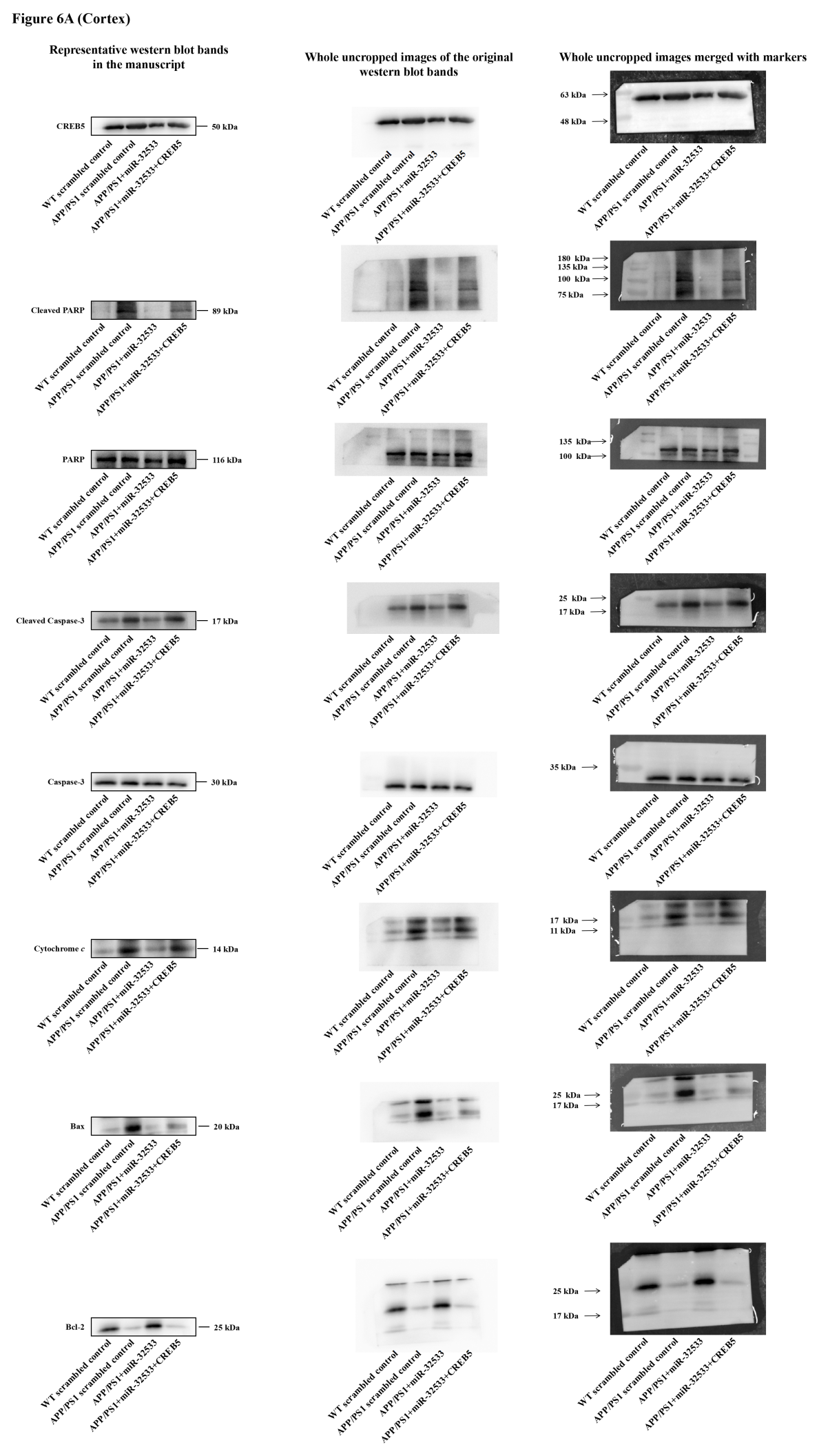

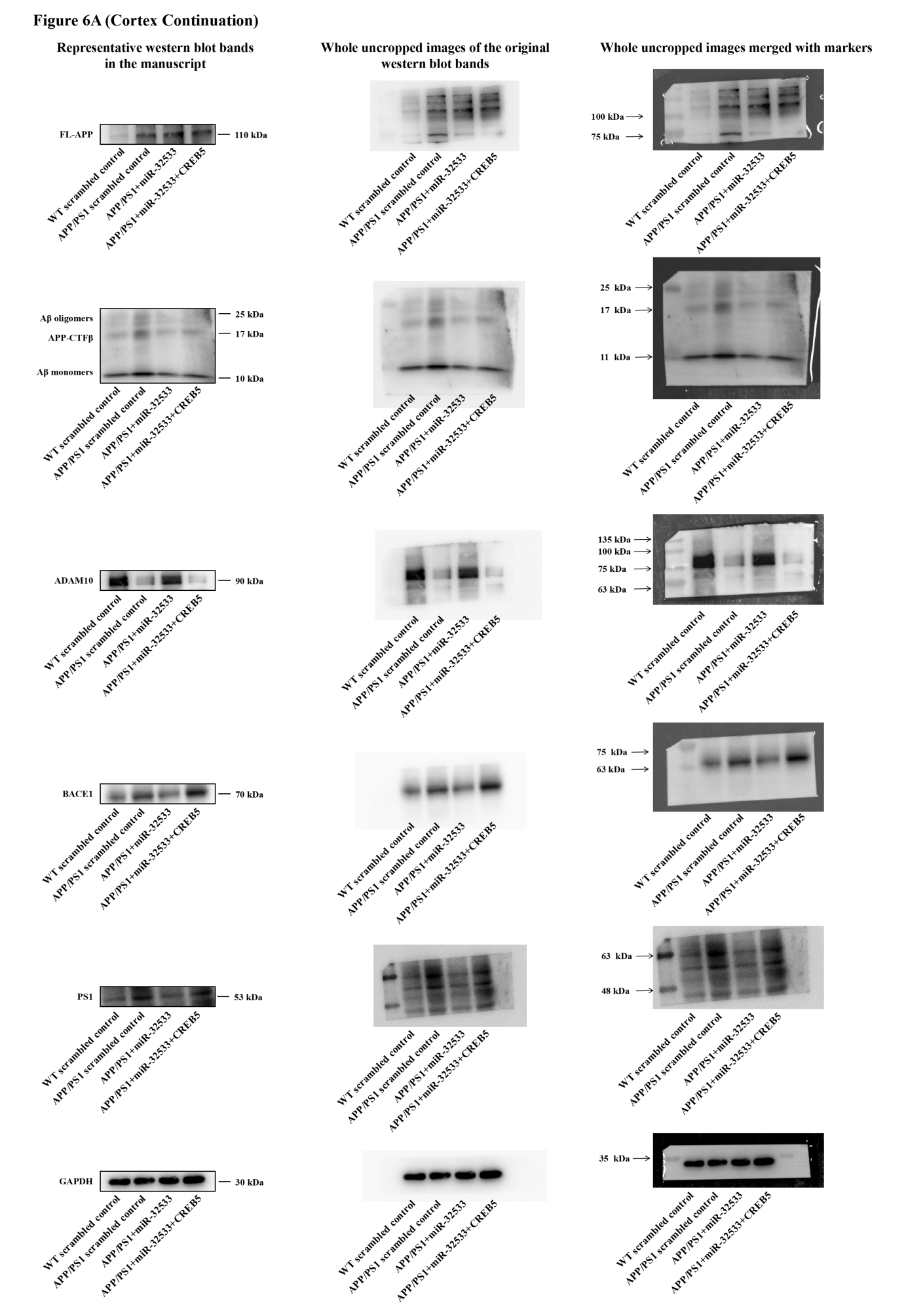


**Figure S20.** Whole uncropped images of the original blots of Figure 6A.


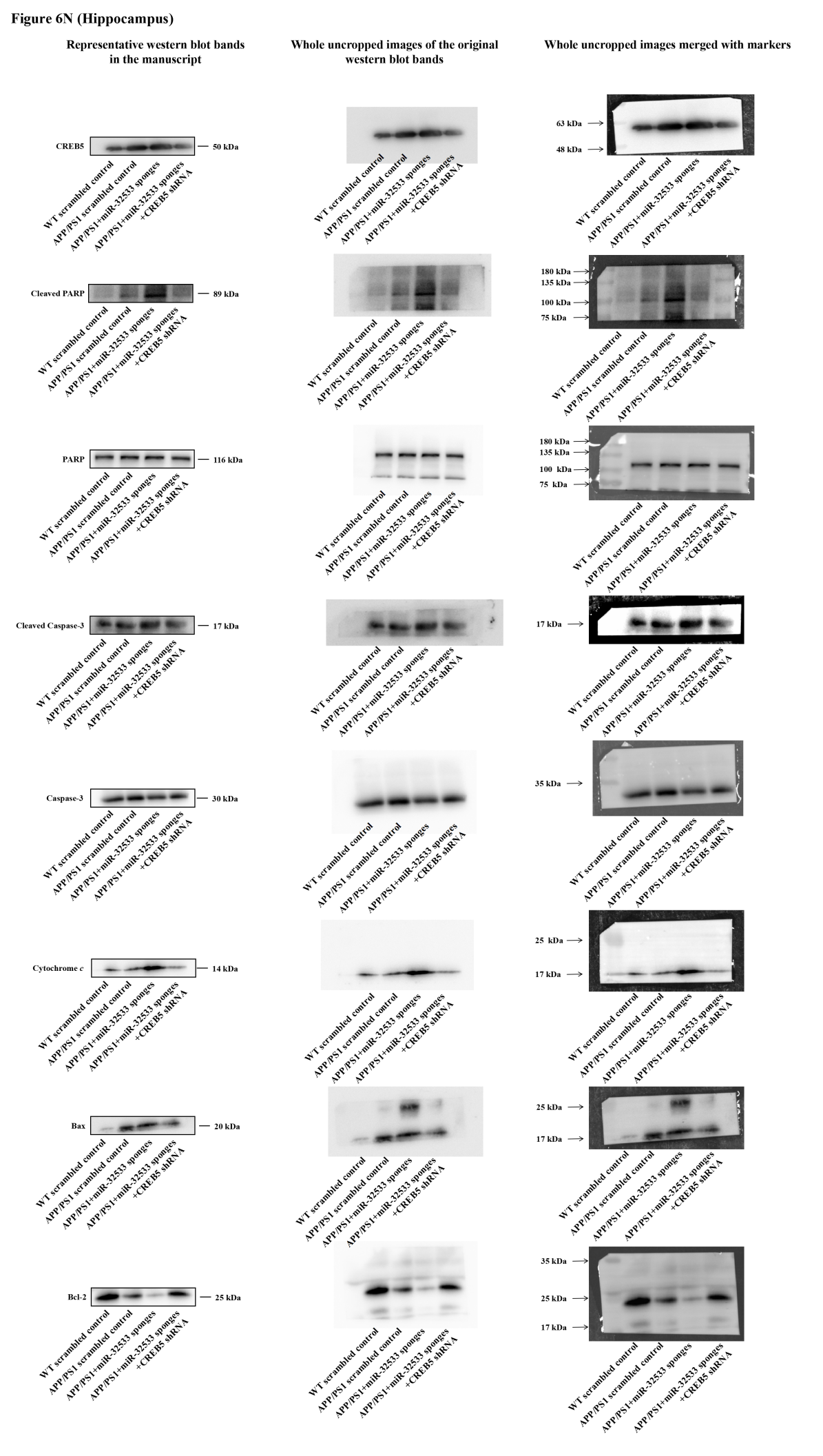

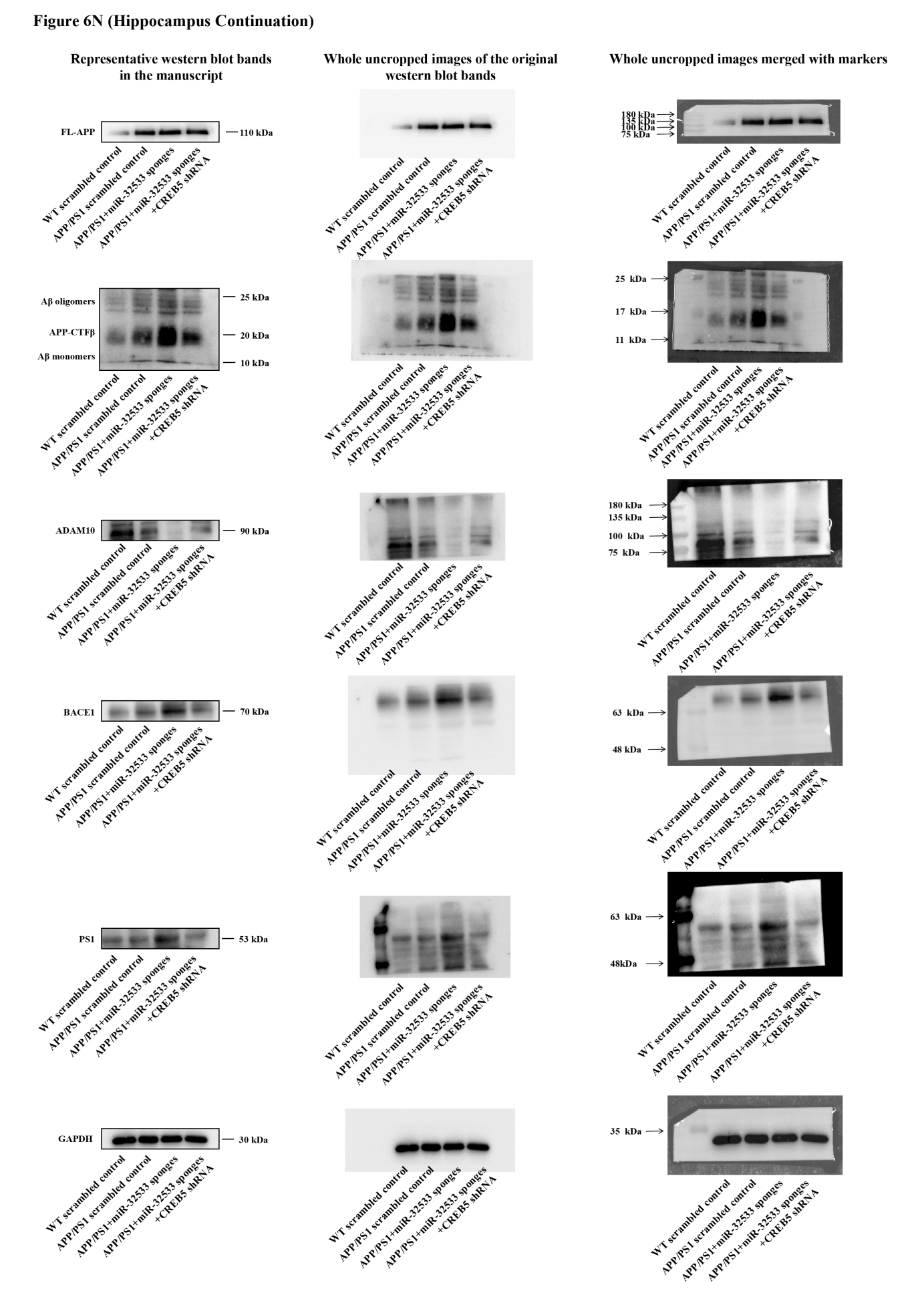

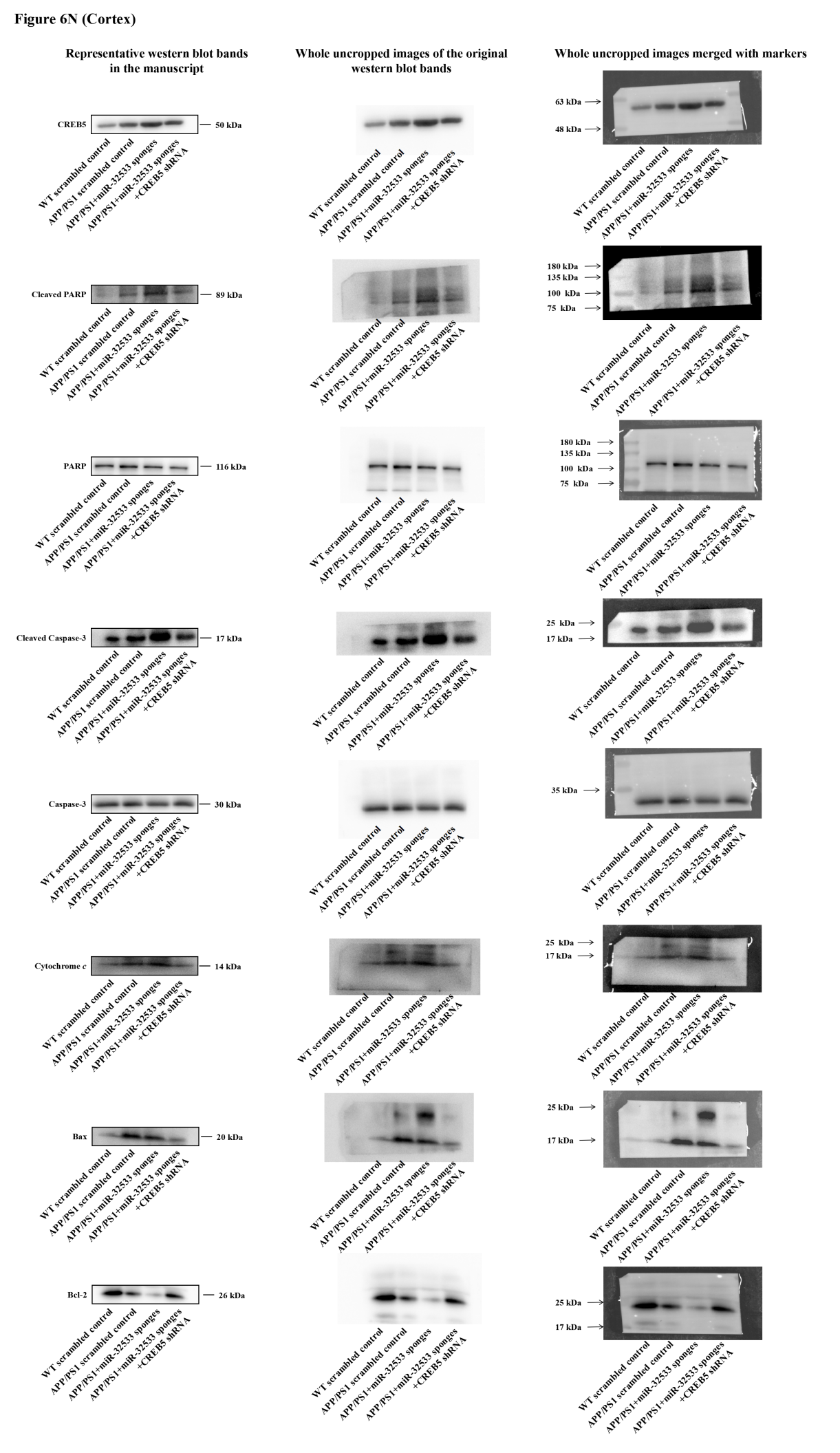

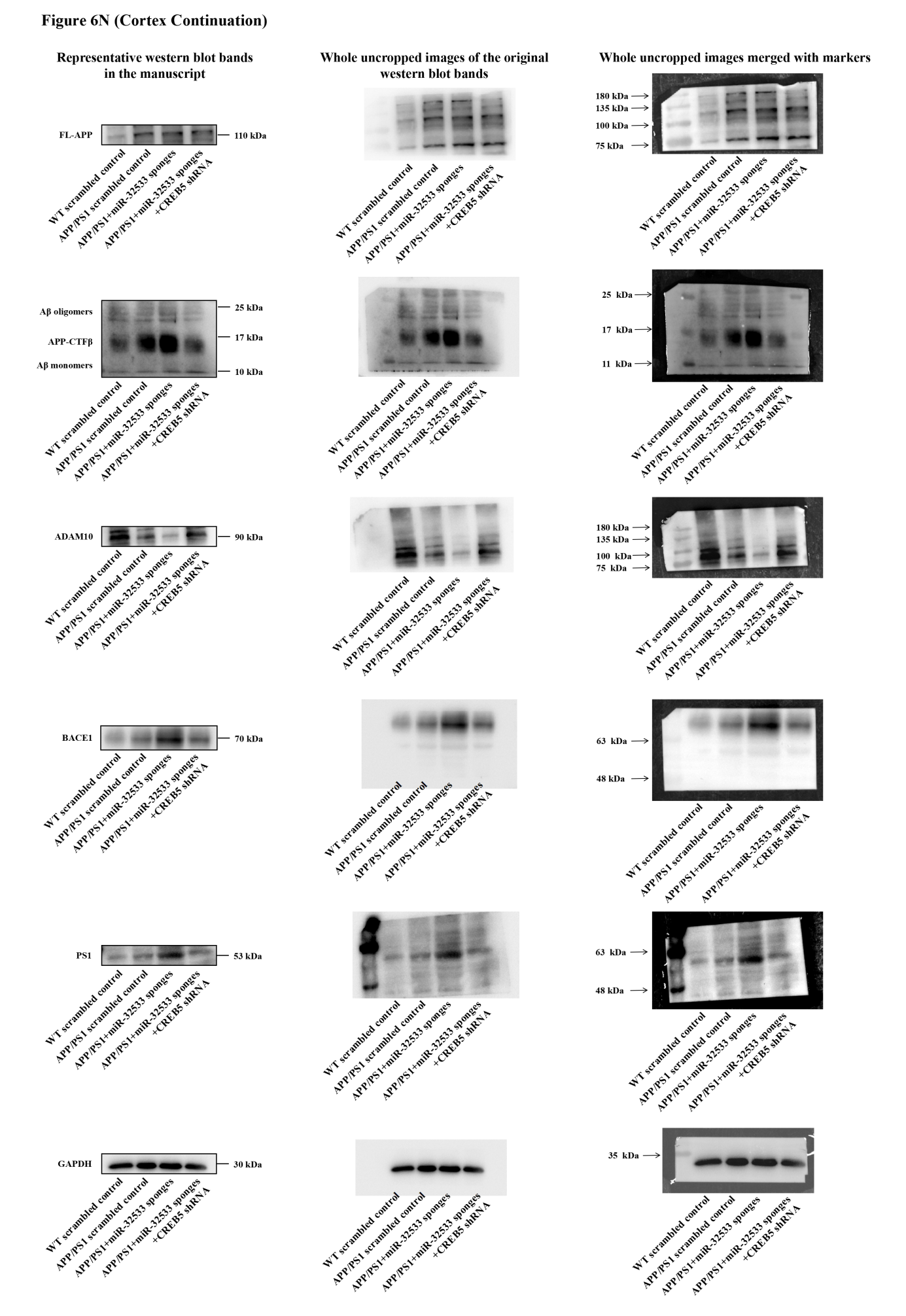


**Figure S21.** Whole uncropped images of the original blots of Figure 6N.
